# Cost-Effectiveness Analysis of Clesrovimab for Respiratory Syncytial Virus in Infants in the United States

**DOI:** 10.3390/vaccines14050411

**Published:** 2026-05-01

**Authors:** Klodeta Kura, John C. Lang, Dawei Wang, Yoonyoung Choi, Michelle G. Goveia, Anushua Sinha, Yao-Hsuan Chen, Elamin H. Elbasha

**Affiliations:** 1 Health Economic and Decision Sciences, MSD (UK) Limited, London EC2M 6UR, UK; yao-hsuan.chen@msd.com; 2 Health Economic and Decision Sciences, Merck Canada Inc., Kirkland, QC H9H 4M7, Canada; john.lang@merck.com; 3 Health Economic and Decision Sciences, Merck & Co., Inc., Rahway, NJ 07065, USA; dawei.wang@merck.com (D.W.); elamin_elbasha@merck.com (E.H.E.); 4 Epidemiology, Merck & Co., Inc., Rahway, NJ 07065, USA; yoon.young.choi2@merck.com; 5 Global Medical and Scientific Affairs, Merck & Co., Inc., Rahway, NJ 07065, USA; michelle_goveia@merck.com; 6 Global Clinical Development, Merck & Co., Inc., Rahway, NJ 07065, USA; anushua.sinha@merck.com

**Keywords:** RSV, respiratory syncytial virus, clesrovimab, modeling, cost-effectiveness

## Abstract

**Background/Objectives**: Respiratory syncytial virus (RSV) causes a significant hospitalization burden in infants. The objective of this study was to evaluate the cost-effectiveness of introducing clesrovimab, a recently approved long-acting monoclonal antibody, in all US infants born during or entering their first RSV season. **Methods**: A decision analytical model simulated the clinical and economic impact of clesrovimab in a yearly birth cohort, compared with three alternative interventions: nirsevimab, palivizumab, and the RSVpreF maternal vaccine. Model inputs were obtained from the published literature. Efficacy estimates for clesrovimab were derived from post hoc analyses of randomized control trial data, which were conducted to align endpoints from different studies (nirsevimab and RSVpreF). Medically attended lower respiratory infection (MALRI), quality-adjusted life years (QALYs, 3% discounting), and costs (in 2024 USD) were evaluated from a societal perspective. Both deterministic and probabilistic sensitivity analyses were performed. **Results**: Clesrovimab resulted in fewer (38,252) RSV-related MALRI outcomes and was cost-saving compared to nirsevimab, with significant reductions in total costs (USD 98 million saved). When compared with palivizumab, clesrovimab and nirsevimab were estimated to cost USD 38,655 and USD 79,912 per QALY, respectively. Results were sensitive to changes in intervention costs, efficacy, and QALY loss due to RSV infection. **Conclusions**: Clesrovimab may significantly reduce the burden of RSV among US infants in their first RSV season and may save costs compared to nirsevimab.

## 1. Introduction

Human respiratory syncytial virus (RSV) is a seasonal virus in the United States (US) with a typical season occurring between late autumn and early spring [1,2]. RSV usually causes mild lower respiratory tract infections (LRTIs), but can also lead to serious illness or even death in infants and young children (aged less than five years), and it is the most common cause of hospitalization in infancy [3].

While premature infants and children with comorbidities, such as hemodynamically significant congenital heart disease (CHD), bronchopulmonary dysplasia, and Down syndrome, are at higher risk of RSV hospitalization, the majority of RSV hospitalizations occur in healthy, full-term infants [4].

In the US, RSV infection leads to over 57,000 hospitalizations and approximately two million outpatient visits annually among children under five years old [5]. The economic impact of RSV disease in children under five years old in the US is substantial, with estimated direct costs ranging from USD 652 million (2002 US Dollars [USD]) to USD 1.2 billion (2021 USD). Hospitalizations account for the largest proportion of costs, amounting to USD 342 to 530 million, followed by physician office visits (USD 140–220 million) and emergency department (ED) visits (USD 65–215 million) [6,7,8,9].

Strategies to address the substantial burden of RSV disease in infants include immunoprophylaxis and maternal vaccination (MV). Historically, palivizumab has been the primary intervention, administered as up to five monthly injections during the RSV season (recommended for infants at high risk for severe RSV disease only) [10]. In August 2023, the Advisory Committee on Immunization Practices (ACIP) recommended nirsevimab, a long-acting monoclonal antibody, for infants under 8 months entering their first RSV season and for infants at high risk for severe RSV disease up to 19 months [11,12]. In September 2023, the ACIP recommended a bivalent RSV prefusion F protein-based (RSVpreF) vaccine for pregnant women at 32–36 weeks of gestation, with seasonal administration to prevent RSV-associated LRTIs in infants aged less than six months [13].

Clesrovimab is a long-acting human monoclonal antibody that targets the RSV fusion protein, providing durable protection with a single dose. It provides robust protection against mild, moderate, and severe RSV disease for all infants, including full-term, preterm, and those with risk factors for severe RSV disease. Furthermore, efficacy is observed through 6 months for all exploratory endpoints [14,15]. In June 2025, clesrovimab was approved by the U.S. Food and Drug Administration, and the Advisory Committee on Immunization Practices recommended clesrovimab for the prevention of RSV lower respiratory tract disease in all infants who are born during or entering their first RSV season [16].

This study employed a decision analytical model to evaluate the impact of introducing clesrovimab in US infants by comparing clinical outcomes and costs with current recommendations. Previous US cost-effectiveness analyses have assessed the impact of RSV prevention strategies in infants, including comparing clesrovimab to palivizumab, nirsevimab to palivizumab, RSVpreF vaccine to no vaccination, and nirsevimab to clesrovimab [17,18,19,20]. To the authors’ knowledge, this study is the first comprehensive US economic evaluation to directly compare clesrovimab with nirsevimab, palivizumab, and the maternal RSVpreF vaccine. By bringing these interventions together in one model and presenting extensive scenarios and sensitivity analyses, this work provides a unified assessment of their potential clinical and economic outcomes in the US.

## 2. Methods

### 2.1. Perspective

This study’s analysis was conducted from a US societal perspective. The healthcare sector perspective was considered in scenario analysis.

### 2.2. Eligible Patient Population

The analysis included all infants, including preterm and full-term infants, as well as infants at increased risk for severe RSV disease, such as those with CHD and/or chronic lung disease (CLD). The eligible population was divided into 6 sub-groups based on risk status and gestational age: infants with CHD and CLD, non-high-risk infants born full-term (≥37 weeks gestational age [wGA]), late preterm (≥35–<37 wGA), moderately preterm (≥32–<35 wGA), very preterm (≥29–<32 wGA), and extremely preterm (<29 wGA). The population was stratified by gestational age and high-risk status (CHD/CLD) because RSV incidence, severity, and healthcare utilization vary substantially across these strata, with risk increasing as gestational age decreases and among infants with various underlying medical conditions. These categories aligned with clinical trial stratification and with current prevention policies and recommendations, which define eligibility and use of RSV interventions (i.e., palivizumab) according to gestational age and among infants at high risk for severe RSV disease [10].

### 2.3. Intervention Strategies

The populations eligible for clesrovimab and nirsevimab differed from those eligible for palivizumab and for the RSVpreF maternal vaccine. Clesrovimab and nirsevimab were assumed to be given seasonally to infants under 8 months (including preterm and full-term infants, as well as infants at increased risk for severe RSV disease, such as those with CHD and/or CLD) as they entered their first RSV season, either at birth or within the first month of the season (October to March). Palivizumab was administered to extremely preterm infants (<29 wGA) and infants at increased risk for disease (with CHD and/or CLD), with a maximum of five monthly doses. The RSVpreF vaccine was assumed to be administered to pregnant individuals expected to give birth during the RSV season.

### 2.4. Time Frame and Analytic Horizon

The study’s time frame covered twelve months after RSV intervention administration. Infants born before the RSV season were followed from its start (October) until the next season, while those born during the season were followed until their first birthday. The model also tracked RSV-related outcomes that may extend beyond the twelve-month period, such as death, applying a lifetime horizon for these outcomes.

### 2.5. Discounting and Cost Year

Quality-adjusted life years (QALYs) were discounted at 3% annually. All costs were reported in 2024 USD and were inflated using the medical care component of the consumer price index (CPI) from the US Bureau of Labor Statistics [21].

### 2.6. Economic Model Structure

**Figure 1:**
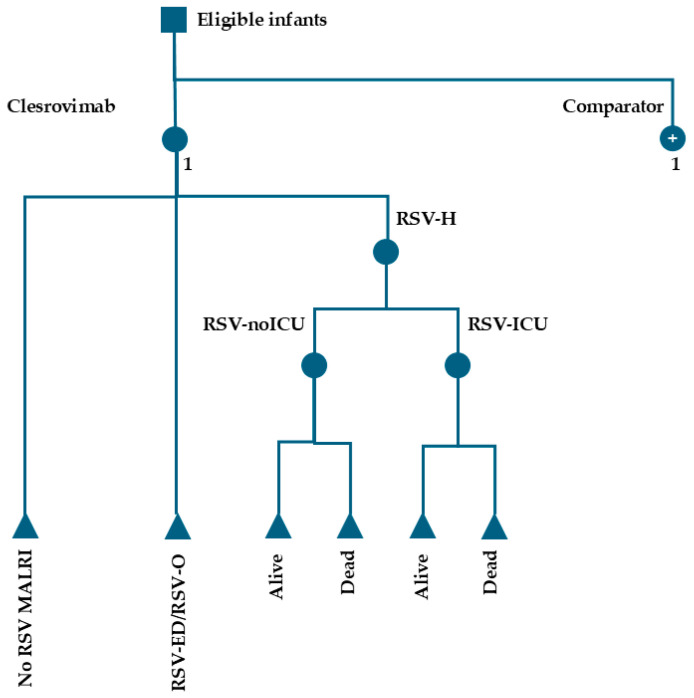
Decision analytical model structure.

A decision analytical model was developed in Microsoft^®^ Excel 365 (Microsoft Corporation, Redmond, WA, USA) to simulate a US birth cohort’s experience in the twelve months following administration of the RSV intervention, covering their first RSV season (Figure 1) [22]. Infants could become infected with RSV and require medical attention (medically attended lower respiratory infection [MALRI]) in either an outpatient setting (such as an emergency department visit [RSV-ED] or a physician’s office visit [RSV-O]) or an inpatient setting (hospitalization [RSV-H]). A proportion of RSV-H cases could require admission to an intensive care unit (RSV-ICU; those without ICU admission were referred to as RSV-noICU), and a portion of RSV-H cases could result in death.

Abbreviations: MALRI, medically attended lower respiratory infection; RSV, respiratory syncytial virus; RSV-ED, RSV emergency department visit; RSV-H, RSV hospitalization with or without intensive care unit admission; RSV-ICU, RSV-H with intensive care unit admission; RSV-noICU, RSV-H without intensive care unit admission; RSV-O, RSV physician’s office visit. Squares indicate decision nodes; filled circles indicate chance nodes; plus signs denote clone nodes representing identical downstream pathways.

### 2.7. Key Risk Factors: Chronological Age (CA), Gestational Age (GA), and RSV Seasonality

Several studies have evaluated the impact of gestational age (GA) and chronological age (CA) on RSV burden [23,24,25]. However, most published disease burden estimates do not simultaneously account for GA, CA, and varying RSV-related health risk based on the CA of infants as they age through the RSV season. This could potentially lead to less accurate estimates of RSV-related disease burden among infants. For example, because the risk of RSV decreases with either increasing CA or GA at birth, the risk of an RSV-H event differs for infants with a CA of 3 months and <29 wGA versus 6 months CA and ≥37 wGA at the start of the RSV season. This risk also changes based on exposure timing as determined by the month of birth relative to the RSV season. To account for all of this, the risk of disease was allowed to vary by CA, GA, and time of the year.

### 2.8. Model Outcomes

This analysis focused on RSV MALRI, excluding RSV non-LRTIs. The model outcomes included RSV-H (with and without ICU admission), RSV-ED, RSV-O, and RSV-associated deaths, with and without interventions. It also estimated QALYs, while health and economic outcomes averted between comparators were summarized using the incremental cost-effectiveness ratio (ICER). Additionally, the model calculated the number needed to immunize (NNI, following a model-based approach) with clesrovimab to prevent an RSV MALRI outcome of interest.

## 3. Model Inputs

The main inputs are described in Table 1, including (i) the population at risk, (ii) RSV seasonality, (iii) the incidence and cost data of RSV-H, RSV-ED, and RSV-O, (iv) quality of life data, and (v) efficacy data for each intervention. Details of the main and other inputs used in this study are described in the Supplementary Materials (Sections S1–S3; Tables S1–S9).

The cost per dose (Table 1) for nirsevimab, RSVpreF, and clesrovimab in this study was based on the weighted average of 2025 list prices and the Vaccines for Children (VFC) price, assuming that 50% of doses would be obtained through the VFC program. Clesrovimab’s cost was assumed to be USD 485 per dose, calculated as the average of the list price (USD 556) and the assumed VFC price of USD 414 (which falls within the potential VFC range of USD 365 to USD 425 per dose). Administration costs were excluded from this analysis.

### Efficacy and Coverage Rate of Interventions

To estimate the impact of each intervention on the incidence of RSV MALRI and the associated costs, the efficacy of each intervention, its coverage rate (CR), and the duration of protection were considered. Efficacy was defined as the proportion of MALRI cases prevented by the intervention. Efficacy included two properties. The first property was the initial efficacy of the intervention. The second property was the waning of protection, i.e., the reduction of efficacy over time. CR was defined as the proportion of eligible infants who receive the intervention (or who were impacted by the intervention in the case of maternal vaccine) and complete its full regimen. It was assumed that coverage was the same whether the infant was born off-season or during the season. The coverage rate for each intervention was assumed to be 50%. Finally, the duration of protection was defined as the amount of time for which an intervention’s efficacy was positive. Data on efficacy and duration of protection for comparators were obtained from randomized clinical trials [RCTs].

Nirsevimab efficacy estimates against MALRI and against RSV-H were estimated from pooled phase 2b and phase 3 RCT data; however, efficacy data against RSV-ED and RSV-O were not presented [41]. Given the lack of granularity in the efficacy data and the timeframe of the clinical trial, the MALRI efficacy for nirsevimab (79% for <6 months post-dose) was applied equally to all outcomes (i.e., RSV-H, RSV-ED, and RSV-O) [12,41,43]. To ensure consistency between comparisons, for each intervention, MALRI efficacy was applied equally to all outcomes.

The MALRI efficacy of the RSVpreF vaccine in full-term infants was estimated by RCTs to be 51.3% over a 6-month period. It should be noted that the efficacy of the RSVpreF vaccine for preterm infants and infants at high risk for severe RSV disease was extrapolated by following the methodology of Rainisch et al. (2020) [22,30].

Palivizumab’s efficacy was 51.0% for all MALRI outcomes (i.e., RSV-H, RSV-ED, and RSV-O) for infants at high risk for severe RSV disease (infants born <29 wGA or with CHD/CLD) [39]. The duration of protection for palivizumab was assumed to be 1 month, i.e., it was assumed that palivizumab protected infants only during the months in which it was administered. Additionally, it was assumed that palivizumab was administered during the RSV season up to a maximum of five administrations, i.e., for a maximum duration of protection of 5 months, with a constant coverage across the season [10].

Evaluating the impact of clesrovimab versus a specific comparator requires proper alignment between the endpoints used in the respective RCTs. Because different RCTs for nirsevimab, the RSVpreF vaccine, and clesrovimab used different case definitions and endpoints, endpoints were harmonized across trials to permit meaningful comparisons. Below is a summary of the efficacy inputs used when evaluating the impact of clesrovimab against nirsevimab, the RSVpreF vaccine, and palivizumab (for infants at high risk of severe RSV disease).

*Clesrovimab versus Nirsevimab:* The primary efficacy endpoint in the clinical trials of clesrovimab compared with placebo was MALRI ≥ 1 indicator of LRI or disease severity. However, clesrovimab’s endpoint that most closely aligned with the primary efficacy endpoint of medically attended RSV LRTI in the clinical efficacy trials of nirsevimab was MALRI requiring ≥2 indicators of LRI/severity (87.2% through 6 months post-dose) [14,15]. Details on the methods and diagnostic criteria for each RSV-associated endpoint are described in Zar et al. (2025) [14,15].

In comparison 1 (Table 2), a constant efficacy of 87.2% for all gestational ages and for all MALRI outcomes (e.g., RSV-H, RSV-ED, and RSV-O) for days 1–180 (equivalent to six months’ duration) post-dose was applied. It was assumed that clesrovimab’s efficacy was 0% after 180 days (i.e., after 6 months). Nirsevimab was modeled with constant MALRI efficacy for the first 5 months and 0% thereafter, similar to methods used in prior cost-effectiveness studies [44,45,46,47,48]. The assumed duration of protection, 6 months for clesrovimab and 5 months for nirsevimab, reflected the maximum follow-up time reported in the respective global registrational RCTs evaluating MALRI.

Two additional scenarios were evaluated: (1) equal 5-month durations of protection for both interventions, and (2) an alternative waning function for post-trial protection.

*Clesrovimab versus the RSVpreF vaccine:* Aligning the endpoint for clesrovimab efficacy to that used for the RSVpreF vaccine resulted in an efficacy estimate of 75.1% for days 1–180 (i.e., for months 1–6) post-dose. Like the comparison of clesrovimab versus nirsevimab, clesrovimab efficacy was not stratified by GA or by clinical outcome. It was assumed that the efficacy of clesrovimab and the RSVpreF vaccine were 0% after 180 days (i.e., after 6 months).

*Clesrovimab versus palivizumab:* To evaluate the impact of clesrovimab against palivizumab (for infants at high risk of severe RSV disease), the clesrovimab efficacy for MALRI requiring ≥2 indicators of LRI/severity was adopted (comparison 2). This definition most closely aligned with the endpoint used in the study for nirsevimab (and used in previous ACIP evaluations of nirsevimab). It was assumed that clesrovimab’s efficacy was 0% after 180 days (i.e., after 6 months).

## 4. Comparisons: Interventions and Comparators

In this study, three types of comparisons were conducted, as shown in Table 2. Each comparison was conducted within the respective eligible population. For instance, in the comparison between clesrovimab and palivizumab, clesrovimab coverage was applied to 50% of the eligible population, encompassing both high-risk and non-high-risk infants. By comparison, palivizumab coverage was limited to 50% of infants at high risk of severe RSV disease, while the remaining infants were assumed to receive no prophylactic intervention.

### 4.1. Sensitivity Analysis for Comparison #1

Both deterministic (DSA) and probabilistic sensitivity analyses (PSA) were conducted for comparison #1. A full list of parameters included in the sensitivity analyses, as well as a detailed description of the protocol for the DSA and PSA, is included in Table S9 of the Supplemental Materials. Each parameter in the analysis was assigned a probability distribution, and the 2.5% and 97.5% quantiles were computed. In the DSA, parameters were varied sequentially, with the model evaluated at the lower (2.5%), mean (base case), and upper (97.5%) quantiles, while holding other parameters constant. DSA results are shown in tornado diagrams, displaying the impact of the ten most sensitive parameters on ICER. In the PSA, parameters were varied jointly across 1000 pseudorandom realizations, assuming independence between distributions. PSA results are presented using cost-effectiveness acceptability curves. The cost-effectiveness acceptability curve shows the percentages of cost-effective simulation runs on the y-axis and the willingness to pay (WTP) threshold (varying from USD 0 to USD 300,000) on the x-axis. Sensitivity analyses for comparisons #2 through #4 are provided in Section S4 of the Supplementary Materials.

### 4.2. Scenario Analysis for Comparison #1

The following scenarios were analyzed to evaluate how the quantitative and qualitative results changed for comparison #1.

#### 4.2.1. Scenario 1.1 (S1.1): Alternative Waning and Durations

In this scenario, an alternative waning assumption was adopted for the efficacy beyond the time frame obtained in RCTs. In particular, a linear decay was assumed from month 6 to month 10 for clesrovimab and from month 5 to month 10 for nirsevimab, respectively (reaching 0% efficacy at the end of month 10 for both interventions). The assumption of a 10-month duration of protection was consistent with that used by Hutton et al. (2024) [18].

#### 4.2.2. Scenario 1.2 (S1.2): Alternative Duration of Protection

In this scenario, clesrovimab and nirsevimab were both assumed to provide a 5-month duration of protection. For clesrovimab, efficacy over the first 5 months post-dose was 88% (MALRI requiring ≥2 indicators of LRI/severity).

#### 4.2.3. Scenario 2 (S2): Alternative Cost Data

This scenario considered alternative data for RSV-H cost and percentage admitted to the ICU (see Section S3 of the Supplementary Materials).

#### 4.2.4. Scenario 3 (S3): Healthcare Sector Perspective

In this specific scenario, the healthcare sector analytical perspective was adopted [49].

## 5. Results

### 5.1. Comparison #1 Analysis

The overall clinical impact of the clesrovimab intervention compared to the nirsevimab intervention is summarized in Table 3. Compared to nirsevimab, clesrovimab prevented approximately 26,268 outpatient cases, 9193 ED visits, and 2787 hospitalizations annually, and averted three RSV-related deaths. The decrease in hospitalizations included both non-ICU and ICU admissions, reducing demand for inpatient beds and critical care resources during peak RSV months. Economically, total intervention costs were the same across both interventions, but clesrovimab reduced treatment costs and produced savings of about USD 98 million. Most of these savings were derived from avoided hospital care, which constituted the largest component of RSV treatment costs in the model. When both interventions were priced at USD 485 per dose, clesrovimab was cost-saving versus nirsevimab (see Table 3 for the ICER and full cost breakdown).

### 5.2. Comparison #2 and Comparison #3 Analysis

In comparison #2 (clesrovimab vs. palivizumab) and comparison #3 (nirsevimab vs. palivizumab), clesrovimab reduced RSV MALRI outcomes by 217,153 compared to palivizumab, while nirsevimab achieved a reduction of 178,901 (Table 3). The ICER for clesrovimab versus palivizumab in comparison #2 was USD 38,655/QALY. For nirsevimab versus palivizumab in comparison #3, the ICER was USD 79,912/QALY.

When comparing clesrovimab and nirsevimab to palivizumab, the NNI was consistently lower for clesrovimab (see Table S10), indicating greater impact. Specifically, to prevent one RSV outpatient visit, 15 individuals needed to be immunized with nirsevimab versus 13 with clesrovimab. To prevent one RSV emergency department visit, the NNI was 42 for nirsevimab compared to 34 for clesrovimab. To prevent an RSV hospitalization, 122 people needed to be immunized with nirsevimab versus 103 with clesrovimab. It should be noted that NNI was expected to differ between comparators due to different efficacy values.

### 5.3. Comparison #4 Analysis

In comparison #4 (clesrovimab versus the RSVpreF vaccine), clesrovimab produced larger reductions in all RSV outcomes than the maternal RSVpreF vaccine, but did so at a higher total cost (driven by differences in assumed program costs), yielding an ICER of USD 124,325/QALY (Table 4).

### 5.4. Sensitivity Analysis for Comparison #1

#### 5.4.1. One-Way Deterministic Sensitivity Analysis

**Figure 2:**
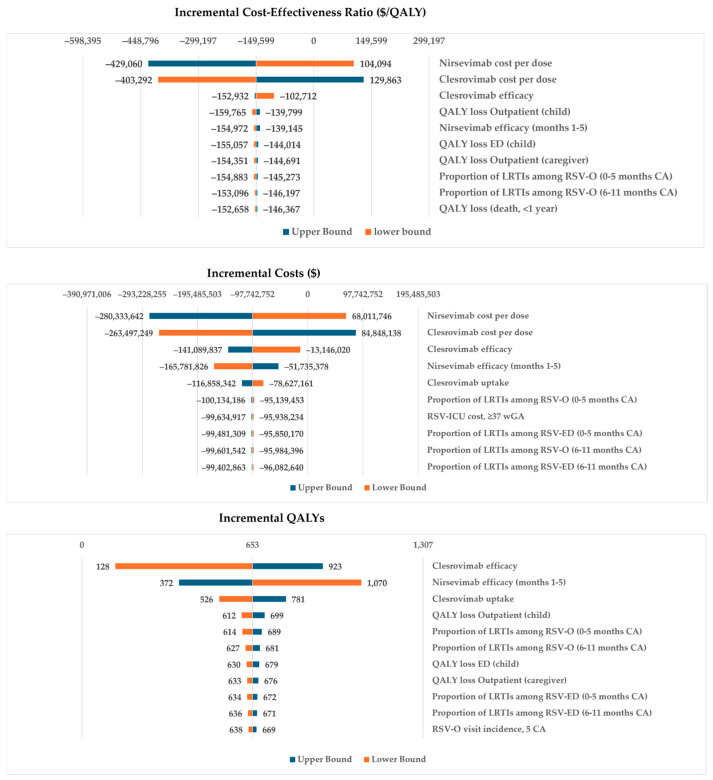
DSA of clesrovimab versus nirsevimab showing the most influential inputs on the ICER (Top), Incremental costs (Middle), and Incremental QALYs (Bottom). Abbreviations: CA, chronological age; LRTI, lower respiratory tract infection; QALY, quality-adjusted life-year; ED, RSV emergency department visit.

Figure 2 presents the one-way DSA results for comparison #1, evaluating clesrovimab and nirsevimab, both priced at USD 485 per dose. The key factors influencing the ICER were intervention cost-per-dose, efficacy, and QALY loss due to RSV.

#### 5.4.2. Probabilistic Sensitivity Analysis

**Figure 3:**
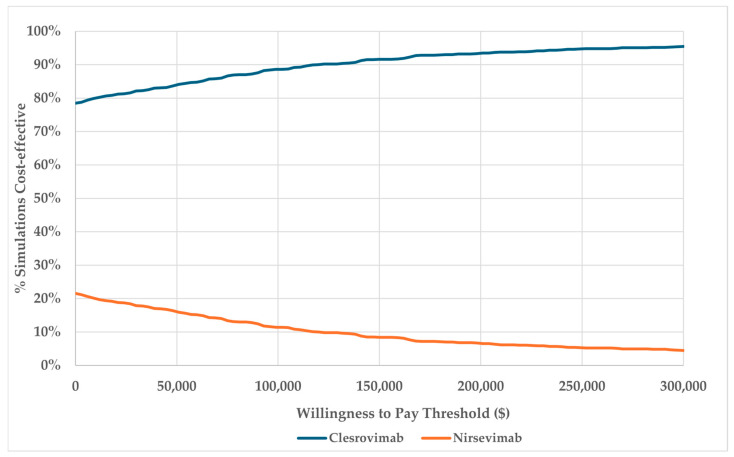
Cost-effectiveness acceptability curve for clesrovimab versus nirsevimab (the proportion of PSA iterations in which the clesrovimab intervention is cost-effective versus the nirsevimab intervention).

Figure 3 presents the PSA results for comparison #1, which evaluates clesrovimab and nirsevimab at a price of USD 485 per dose. The analysis showed that 78.5% of PSA simulations were cost-saving, and 88% had an ICER below USD 100,000.

#### 5.4.3. Scenario Analysis for Comparison #1

In scenarios 1.1 and 1.2, whether applying an alternative waning function or assuming the same protection duration for both treatments, clesrovimab remained cost-saving when compared with nirsevimab (see Table 5). The base case results were also robust to alternative cost and healthcare data (scenario 2), where RSV-H costs were higher and RSV-ED/RSV-O costs were lower compared to the base case, with lower RSV-ICU admission rates. Scenario 3 also found clesrovimab to be cost-saving from a healthcare sector perspective.

## 6. Discussion

A decision analytical model was used to assess the healthcare and economic impact of clesrovimab in US infants over 12 months, from a societal perspective (base case, healthcare sector perspective is explored in scenario analysis). The model accounted for gestational age (GA), birth month, and chronological age (CA), along with monthly RSV incidence variations, improving the accuracy of clinical outcomes. Efficacy estimates for the interventions were based on clinical trial data (from the global registrational clinical trial data evaluating MALRI). Constant efficacy was assumed for 6 months with clesrovimab and 5 months with nirsevimab, followed by either (i) a drop to zero or (ii) a linear decay.

Compared to nirsevimab, clesrovimab reduced RSV MALRI outcomes by 9.5%, with the greatest impact and cost savings seen in healthy full-term infants. Clesrovimab was also found to be cost-saving compared to nirsevimab, with sensitivity analyses confirming robustness (clesrovimab was cost-saving in 78% of PSA runs).

Scenario analyses supported these findings: when alternative waning assumptions are applied beyond RCT follow-up, i.e., when efficacy declined linearly to 0% by month 10 and when both interventions were assumed to provide a uniform 5-month duration of protection, the results still favored clesrovimab; updated cost/resource use resulted in higher RSV-H but lower RSV-ED/RSV-O costs; and cost savings were confirmed when the analysis was conducted from a healthcare sector perspective.

In comparison to palivizumab, clesrovimab reduced RSV MALRI by 37.5% (vs. 30.9% for nirsevimab compared to palivizumab), with ICERs of USD 38,655/QALY for clesrovimab and USD 79,912/QALY for nirsevimab. Against RSVpreF, clesrovimab’s ICER was USD 124,325/QALY. For this comparison, two contextual points are important. First, clesrovimab was predicted to prevent more RSV MALRI outcomes than RSVpreF. Second, the higher ICER was primarily driven by differences in assumed program costs and may still be considered acceptable depending on policy perspective and decision criteria. Notably, there is no formally adopted cost-effectiveness threshold in the United States. However, several organizations have published reference ranges to inform decision-making. For example, the Institute for Clinical and Economic Review (ICER) commonly cites benchmark ranges of approximately USD 100,000–150,000 per QALY gained, depending on context, disease severity, and uncertainty, which are often used as decision aids rather than strict thresholds [50,51].

Previous modeling conducted by the University of Michigan and the CDC team (UM-CDC), presented during the ACIP meetings, assessed the cost-effectiveness of nirsevimab and clesrovimab in comparison to palivizumab. For nirsevimab versus palivizumab, the UM-CDC reported an ICER of USD 153,517/QALY, based on a per-dose cost of USD 445 [18]. For clesrovimab versus palivizumab, the ICER was lower, at USD 104,543/QALY, based on a per-dose cost of USD 457 [52]. These results suggest that, according to the UM-CDC modeling work, clesrovimab was more cost-effective than nirsevimab, even at a higher price point. These findings are consistent with what we found in our study. In this analysis, palivizumab was fully replaced by clesrovimab under the intervention strategy. In contrast, the UM-CDC model assumed that 50% of eligible infants at high risk for severe RSV disease who did not receive clesrovimab still received palivizumab. This led to higher intervention costs for the clesrovimab strategy in the UM-CDC model, as it included the costs of both clesrovimab and palivizumab. This difference in assumptions contributed to the variation in ICER outcomes between the two models.

When comparing the MSD and UM-CDC models, outcomes were found to be most sensitive to factors such as palivizumab coverage rates, the waning of efficacy, the number of palivizumab vials used, and healthcare costs (including ICU, emergency department, and outpatient care).

Recent US modeling studies by Kieffer et al. (2025) and Yarnoff et al. (2026) assessed the impact of clesrovimab versus nirsevimab in infants, using efficacy assumptions that underestimated the benefits of clesrovimab [20,53]. These analyses concluded that nirsevimab would avert more RSV-related events than clesrovimab, which contrasts with the above-presented findings that clesrovimab reduced more RSV MALRI relative to nirsevimab and was cost-saving in the base case (with robustness across multiple sensitivity and scenario analyses). Key differences between this study and Kieffer et al. (2025) and Yarnoff et al. (2026) arise from methodological assumptions [20,53]. Specifically, Kieffer et al. (2025) and Yarnoff et al. (2026) applied shorter durations of protection and inconsistent endpoint definitions for clesrovimab, and mixed real-world effectiveness estimates for nirsevimab with trial-based efficacy for clesrovimab, introducing structural bias [20,53]. In contrast, this analysis consistently applied randomized trial-based efficacy and duration, harmonized endpoint definitions (two indicators of LRI/severity), and aligned waning assumptions across interventions, providing a more accurate evaluation of these interventions. The RCTs included in this study were selected for consistency in trial design, efficacy endpoints, and consideration of global seasonality.

This analysis was parameterized to US epidemiology, healthcare utilization, and unit costs, and therefore its quantitative results may not generalize to other countries where RSV mortality, access to hospitalization, and disease treatment costs, as well as intervention costs, can differ substantially. This may be especially true in low- and middle-income countries (LMICs), where the RSV disease burden is high. Future research is planned to extend this modeling framework to other settings, including LMICs.

### Limitations

One limitation of this model was the lack of data for certain input parameters. For example, there were no data on RSV-O and RSV-ED visits stratified by chronological age (CA) and gestational age (GA), so assumptions were made that these parameters were constant across all GAs and risk categories. Similarly, RSV-H incidence for infants with CHD and CLD was not stratified by GA or CA.

Additionally, the administration costs (including indirect administration costs, e.g., travel) and the impact of adverse events for any intervention were not included (and will be incorporated in future work). These simplifying assumptions are expected to have a limited impact on the results for certain comparisons. For example, clesrovimab and nirsevimab require the same number of administrations per infant. Therefore, under the assumption of similar coverage levels and comparable per-dose administration costs, the latter is unlikely to materially influence the incremental cost estimates between these two interventions. In contrast, the number and cost of administrations differ for the seasonal RSVpreF vaccine and palivizumab compared with nirsevimab or clesrovimab. As a result, the impact of this simplifying assumption may be greater for comparisons involving those interventions and will be examined further in future analyses. Similarly, assuming similar incidence, costs, and QALY loss from adverse events for both mAbs, the impact of excluding adverse events on incremental costs and QALYs is also expected to be small. The efficacy data for nirsevimab and RSVpreF lacked granularity to differentiate between severe (RSV-H) and less severe outcomes (RSV-ED, RSV-O), leading to assumptions that the same efficacy applied to all outcomes. This could result in underestimating averted RSV-H outcomes and overestimating RSV-ED and RSV-O outcomes. More detailed efficacy data would improve model accuracy.

Finally, the analysis focused only on RSV LRTIs, excluding non-LRTIs.

## 7. Conclusions

The findings in this study indicated that implementing clesrovimab among infants in the US could lead to substantial improvements in their health outcomes and in cost-savings relative to nirsevimab. It also demonstrated better cost-effectiveness than nirsevimab relative to palivizumab, with ICERs of USD 38,655/QALY for clesrovimab versus USD 79,912 for nirsevimab. Against the RSVpreF vaccine, clesrovimab’s ICER was USD 124,325/QALY.

## Figures and Tables

**Table 1:** Main model input.

**Part A: Epidemiologic Data**										
**Part A.1: Population at risk (Total population of 3,667,758)**										
**Gestational Age/High-Risk Condition**					**Fraction of Total Population (%)**					**Source**
≥37 wGA					89.68					[18,26]
35–36 wGA					5.95					
32–34 wGA					2.57					
29–31 wGA					0.72					
<29 wGA					0.6					
CHD					0.25					
CLD					0.24					
**Part A.2: RSV Seasonality**										
**Month**					**RSV-Positive Test Results (%)**					**Source**
January					25.00					[27]
February					21.85					
March					13.86					
April					4.51					
May					1.30					
June					0.60					
July					0.47					
August					0.62					
September					1.16					
October					2.95					
November					8.07					
December					19.61					
**Part A.3: Visit-level incidence of RSV-H, RSV-ED, and RSV-O by chronological age**										
**Chronological Age (Months)**	**RSV-H ^a^ (%)**				**RSV-ED (%)**		**RSV-O (%)**			**Source**
<1	1.77				1.96		8.52			[28,29]
1	3.12				6.42		18.79			
2	2.24				7.24		23.42			
3	1.57				10.52		23.26			
4	1.37				11.6		26.5			
5	1.1				7.13		28.92			
6	0.96				8.18		26.47			
7	0.8				5.61		20.72			
8	0.74				5.56		27.78			
9	0.84				5.56		22.72			
10	0.6				4.04		24.17			
11	0.6				5.56		25.81			
**Part A.4: Visit-Level Incidence of Infant RSV-H by Gestational Age**										
**Gestational Age (Weeks)**				**RSV-H Incidence (%)**				**Source**		
≥37				0.75				[28]		
35–36				1.13						
32–34				1.75						
29–31				1.93						
<29				2.49						
**Part A.5: Fraction of RSV-ED and RSV-O Visits That Are LRTIs**										
**Chronological Age (Months)**		**RSV-ED**		**RSV-O**				**Source**		
<6		65%		65%				[30]		
6–11		50%		30%						
>11		50%		30%						
**Part A.6: RSV-H case fatality ratio**										
**High-Risk Condition**				**RSV-H Case Fatality Ratio**				**Source**		
High-risk infants ^c^				0.90%				[2]		
Non-high-risk infants				0.04%						
**Part B: Cost Data**										
**Part B.1: RSV-noICU and RSV-ICU Costs**										
**Gestational Age/High-Risk Condition**		**RSV-noICU Average Costs**		**RSV-ICU Average Costs**				**Source**		
Non-high-risk, <1 year old								[31,32]		
≥37 wGA		USD 9787		USD 31,915						
35–36 wGA		USD 9746		USD 37,693						
32–34 wGA		USD 13,050		USD 37,302						
29–31 wGA		USD 19,328		USD 61,612						
<29 wGA		USD 21,642		USD 57,383						
Non-high-risk, 12–23 months-old										
All GA		USD 13,591		USD 31,313						
High-risk, <1 year old										
CHD		USD 14,476		USD 44,768						
CLD		USD 17,970		USD 56,400						
**Part B.2: RSV-ED and RSV-O Costs**										
**Outcome**		**Medicaid Costs**		**Commercial**				**Average Costs ^b^**	**Source**	
RSV-ED		USD 552		USD 1851				USD 1306	[31,32]	
RSV-O		USD 157		USD 246				USD 209		
**Part B.3: Intervention Costs**										
**Intervention**		**List Price**		**Vaccines for Children Price**				**Weighted Average Price**	**Source**	
Palivizumab		USD 1228/vial		-				USD 1228/vial	[18]	
RSVpreF		USD 306/dose		USD 230/dose				USD 268/dose	[33]	
Nirsevimab		USD 556/dose		USD 414/dose				USD 485/dose	[33], Assumption	
Clesrovimab		USD 556/dose		USD 414/dose				USD 485/dose		
**Part C: Quality of Life Data**										
**Outcome/Chronological age**				**QALYs (in days)**				**Source**		
Infant										
RSV-H				6.2				[18]		
RSV-ED				4.9						
RSV-O				3.1						
Caregiver										
RSV-H				2.4				[18]		
RSV-ED				2.5						
RSV-O				1.5						
RSV-associated premature death (in years)										
<1-year-olds				27.1				[34,35]		
12–23-month-olds				26.2						
**Part D: Indirect Costs**										
**Part D.1: Number of Hours Lost for Caregivers**										
**Outcome**			**Premature infants**			**Full-term infants**		**Source**		
RSV-H			59.2			59.2		[17]		
RSV-ED			20			20		[36,37]		
RSV-O			20			20				
**Part D.2: Lifetime Productivity Lost**										
**Outcome**						**Lifetime Productivity Lost**		**Source**		
RSV-associated death						$1,914,459		[38]		
**Part E: RSV-Intervention Efficacy Data**										
**Intervention**			**Duration**			**MALRI Efficacy % (95% CI)**		**Source**		
Clesrovimab (MALRI requiring ≥2 indicators of LRI/severity) ^d^			≤6 months post-dose ^e^			87.2 (75.1, 93.4)		[14,15]		
Clesrovimab (MALRI comparable to RSVpreF vaccine endpoint)			≤6 months post-dose ^e^			75.1 (59.9, 84.5)				
Palivizumab			≤5 months post-dose ^e^			51 (37, 64)		[39]		
RSVpreF vaccine			≤6 months post-dose ^e^			51.3 (29.4, 66.8)		[40]		
Nirsevimab			≤5 months post-dose ^e^			79 (68.5, 86.1)		[12,41]		

^a^ RSV-ICU incidence is described in the Supplementary Materials. ^b^ The proportion of infants covered by Medicaid and by commercial insurance was assumed to be 41% and 59%, respectively [42]. ^c^ Extremely preterm infants, born at less than 29 wGA, and infants at high risk for severe RSV disease with either CHD and/or CLD. ^d^ MALRI endpoint comparable to nirsevimab endpoint. The primary efficacy endpoint for clesrovimab was respiratory syncytial virus (RSV)–associated medically attended lower respiratory infection (LRI) that resulted in at least one indicator of LRI or disease severity from days 1 to 150 after injection. ^e^ After this duration, efficacy was assumed to be zero.

**Table 2:** Comparison included in this study.

	Intervention	Comparator
Comparison #1	Clesrovimab for all infants entering their first RSV season	Nirsevimab for all infants entering their first RSV season
Comparison #2	Clesrovimab for all infants entering their first RSV season	Palivizumab for infants at high risk for severe RSV disease entering their first RSV season
Comparison #3	Nirsevimab for all infants entering their first RSV season	Palivizumab for infants at high risk for severe RSV disease entering their first RSV season
Comparison #4	Clesrovimab for all infants entering their first RSV season	RSVpreF vaccine for mothers with expected birth during the RSV season

**Table 3:** RSV MALRI outcomes for comparison #1 (clesrovimab versus nirsevimab), comparison #2 (clesrovimab versus palivizumab), and comparison #3 (nirsevimab versus palivizumab).

RSV–Associated Outcome	Outcomes by Intervention			Incremental Outcomes by Comparison (Intervention vs. Comparator)		
	Clesrovimab	Nirsevimab	Palivizumab	Comparison #1 (Clesrovimab vs. Nirsevimab)	Comparison #2 (Clesrovimab vs. Palivizumab)	Comparison #3 (Nirsevimab vs. Palivizumab)
RSV-O visits	244,822	271,090	390,994	−26,268	−146,172	−119,904
RSV-ED visits	89,152	98,345	142,361	−9193	−53,209	−44,016
RSV-noICU visits	20,522	22,515	33,279	−1993	−12,757	−10,764
RSV-ICU visits	8164	8959	13,168	−795	−5004	−4209
No. of RSV-Death	26	29	37	−3	−11	−8
Total QALY loss ^A^	6272	6925	9906	−653	−3634	−2981
Treatment costs (USD)	979,152,524	1,076,895,275	1,560,811,045	−97,742,751	−581,658,521	−483,915,770
Intervention costs (USD)	889,431,315	889,431,315	167,279,659	0	722,151,656	722,151,656
Total Costs (USD)	1,868,583,839	1,966,326,590	1,728,090,704	−97,742,751.58	140,493,135	238,235,886
ICER (USD/QALY)				Cost saving	38,655	79,912

Abbreviations: ICU, intensive care unit; MALRI, medically attended lower respiratory infection; MV, maternal vaccination; RSV, respiratory syncytial virus; RSV-ED, RSV emergency department visit; RSV-H, RSV hospitalization with or without ICU admission; RSV-ICU, RSV-H with ICU admission; RSV-noICU; RSV-H without ICU admission. The first three numeric columns report the absolute outcomes for each intervention strategy (clesrovimab, nirsevimab, and palivizumab) over the modeled time horizon. The three “Incremental outcome” columns show the differences in outcomes between intervention and comparator (intervention minus comparator) for the three comparisons indicated in the table header. Cost values are reported in 2024 USD. ^A^ Entries in this table are rounded to the nearest integer. Negative numbers in the incremental column indicate gains or events saved with the intervention versus the comparator.

**Table 4:** RSV MALRI outcomes for comparisons #4 (clesrovimab versus RSVpreF).

RSV–Associated Outcome	Intervention		Incremental Outcome (Intervention vs. Comparator)
	Clesrovimab	RSVpreF	Comparison #4 (Clesrovimab vs. RSVpreF)
RSV-O visits	265,217	355,153	−89,936
RSV-ED visits	96,577	129,793	−33,216
RSV-noICU visits	22,341	29,656	−7315
RSV-ICU visits	8886	11,841	−2955
No. of RSV-Death	28	39	−11
Total QALY loss ^A^	6797	9132	−2335
Treatment costs (USD)	1,063,749,255	1,425,882,969	−362,133,714
Intervention costs (USD)	889,431,315	237,048,425	652,382,890
Total Costs (USD)	1,953,180,570	1,662,931,394	290,249,176
ICER (USD/QALY)			124,325

Abbreviations: ICU, intensive care unit; MALRI, medically attended lower respiratory infection; MV, maternal vaccination; RSV, respiratory syncytial virus; RSV-ED, RSV emergency department visit; RSV-H, RSV hospitalization with or without ICU admission; RSV-ICU, RSV-H with ICU admission; RSV-noICU; RSV-H without ICU admission. The first two numeric columns report the absolute outcomes for each intervention strategy (clesrovimab and RSVpreF) over the modeled time horizon. The “Incremental outcome” column shows the differences in outcomes between clesrovimab and RSVpreF. Cost values are reported in 2024 USD. ^A^ Entries in this table are rounded to the nearest integer. Negative numbers in the incremental column indicate gains or events saved with the intervention versus the comparator.

**Table 5:** RSV MALRI outcomes for scenarios 1–3.

Scenarios	Nirsevimab		Clesrovimab		Clesrovimab vs. Nirsevimab		
	QALY Loss ^A^	Cost	QALY Loss ^A^	Cost	Inc. QALY	Inc. Cost	ICER
Comparison #1	6925	1,966,326,590	6272	1,868,583,839	653	−97,742,751.58	Cost-saving
S1.1: alternative waning and durations	6562	1,916,111,244	6140	1,849,482,783	422	−66,628,461.39	Cost-saving
S1.2: alternative duration of protection	6925	1,966,326,590	6568	1,908,007,384	375	−58,319,206	Cost-saving
S2: alternative cost data	6925	1,965,905,705	6272	1,868,881,619	653	−97,024,086	Cost-saving
S3: healthcare sector perspective	4931	1,620,396,236	4466	1,555,139,829	465	−65,256,407	Cost-saving

Abbreviations: ICU, intensive care unit; MALRI, medically attended lower respiratory infection; MV, maternal vaccination; RSV, respiratory syncytial virus; RSV-ED, RSV emergency department visit; RSV-H, RSV hospitalization with or without ICU admission; RSV-ICU, RSV-H with ICU admission; RSV-noICU; RSV-H without ICU admission. ^A^ Entries in this table are rounded to the nearest integer. Negative numbers in the incremental column indicate gains or events saved with the intervention versus the comparator.

## Data Availability

The original contributions presented in this study are included in the article/Supplementary Materials. Further inquiries can be directed to the corresponding author.

## Supplementary Materials

The following supporting information can be downloaded at: https://www.mdpi.com/article/10.3390/vaccines14050411/s1, Table S1: Proportion of RSV-H cases admitted to the ICU by GA; Table S2: RSV-H costs per visit (stratified by CA and GA); Table S3: RSV-noICU costs per visit (unstratified by CA); Table S4: RSV-noICU costs per visit (stratified by CA and GA; 2024 USD); Table S5: RSV-ICU costs per hospitalization; Table S6: RSV-ED and RSV-O costs per episode; Table S7: RSV-ICU costs per hospitalization (2024 USD); Table S8: Percentage of RSV-H cases admitted to the ICU; Table S9: Parameter distributions for sensitivity analyses; Table S10: NNI to avert one MALRI outcome; Figure S1: DSA of clesrovimab versus palivizumab showing the most influential inputs on the ICER (Top), Incremental costs (Middle) and Incremental QALYs (Bottom); Figure S2: Cost-effectiveness acceptability curve for clesrovimab versus palivizumab (the proportion of PSA iterations in which the intervention is cost-effective); Figure S3: DSA of nirsevimab versus palivizumab showing the most influential inputs on the ICER (Top), Incremental costs (Middle) and Incremental QALYs (Bottom); Figure S4: Cost-effectiveness acceptability curve for nirsevimab versus palivizumab (the proportion of PSA iterations in which the intervention is cost-effective); Figure S5: DSA of clesrovimab versus maternal vaccine showing the most influential inputs on the ICER (Top), Incremental costs (Middle) and Incremental QALYs (Bottom); Figure S6: Cost-effectiveness acceptability curve for clesrovimab versus maternal vaccine (the proportion of PSA iterations in which the intervention is cost-effective).
