# Supplementary material for: Cost-Effectiveness Analysis of Clesrovimab for Respiratory Syncytial Virus in Infants in the United States
Source: Vaccines (Basel). 2026 May 1;14(5):411. doi: 10.3390/vaccines14050411 (PMC13211542; doi:10.3390/vaccines14050411)
Supplement: Supplementary file 1 [file vaccines-14-00411-s001.zip › vaccines-4263228-supplementary.pdf]

Supplementary Material

S1. Epidemiologic Inputs

1.1 RSV-ICU incidence

RSV-H consisted of hospitalizations with or without an intensive care unit admission (RSV-ICU or RSV-noICU, respectively). The proportion of RSV-H cases who were admitted to the ICU was estimated to be the weighted average of estimates for Medicaid and commercially insured infants (**Table S1**) [1,2]. The proportion of infants covered by Medicaid and by commercial insurance was assumed to be 41% and 59%, respectively [3].

Table S1: Proportion of RSV-H cases admitted to the ICU by GA

GA (wGA)	Fraction of Medicaid RSV-H admitted to ICU	Fraction of Commercial RSV-H admitted to ICU	Average
**Non-high-risk, <1 year old**			
≥ 37	36.7%	21.3%	27.6%
35-36	27.5%	26.6%	26.9%
32-34	32.4%	31.3%	31.8%
29-31	41.8%	32.4%	36.2%
< 29	42.1%	32.5%	36.4%
**Non-high-risk, 12-23 months-old**			
All GA	34.5%	19.8%	25.8%
**High-risk, <1 year old[A]**			
CHD	46.7%	30.3%	37.0%
CLD	41.1%	31.6%	33.5%
**Source**	**[2]**	**[1]**	**Calculated**

Abbreviations: CHD, congenital heart disease; CLD, chronic lung disease; GA, gestational age; ICU, intensive care unit; RSV-H, respiratory syncytial virus hospitalization with or without ICU admission; wGA, weeks gestational age

[A] High-risk 12-23-month-olds were assumed to have the same rate of admission to the intensive care unit as <1-year-olds (due to lack of data).

S2. Cost inputs

2.1 RSV-noICU costs

RSV-noICU Medicaid and commercial costs in 2021 USD were obtained from publications of Choi and colleagues and Tran and colleagues, respectively, and were inflated to 2024 USD (**Table S3**) [1,2,4]. Average (by coverage) RSV-noICU costs

were calculated as a weighted average for births covered by Medicaid (41%) and by commercial insurance (59%) [3]. RSV-noICU cost stratification by CA for non-high-risk infants (i.e., infants without CHD or CLD) was estimated by applying multiplying factors (see **Table S2** and **Table S4**, below, for details). The cost of RSV-noICU for infants with CHD and/or CLD was not stratified by CA or by GA due to a lack of data.

RSV-H Medicaid and commercial costs in 2014 USD that were stratified by chronological age (i.e., for <3-months-olds and <12-months-olds) and gestational age (i.e., < 29, 29-30, 31-32, 33-34, 35-36, and ≥37 wGA) were obtained also from the literature (Table S2) [5]. Costs for 3– to 11-month-olds were estimated by assuming that costs for <12-month-olds were a weighted average of costs for <3-month-olds and costs for 3-11-month-olds (with weights given by the number of observations in each category). Average (by coverage) RSV-noICU costs were calculated as a weighted average of births covered by Medicaid (41%) and by commercial insurance (59%) [3]. The ratio of costs for <3-month-olds to costs versus <12-month-olds and for 3-11-month-olds versus <12-month-olds was calculated.

Table S2: RSV-H costs per visit (stratified by CA and GA)

GA/high-risk condition	Medicaid costs in 2014 USD (Number of observations)			Commercial costs in 2014 USD (Number of observations)			Average costs in 2014 USD (Value relative to <12 months)		
	< 3 months	< 12 months	3-11 months	< 3 months	< 12 months	3-11 months	< 3 months	< 12 months	3-11 Months
**Non-high-risk, <1 year old**									
≥ 37 wGA	$9,567 (12,699)	$8,324 (24,487)	$6,985	$11,562 (7,478)	$10,570 (13,885)	$9,412	$10,744 (1.11)	$9,649	$8,417 (0.87)
35-36 wGA	$15,020 (1,329)	$11,127 (2,521)	$6,787	$21,546 (705)	$16,299 (1,292)	$9,997	$18,870 (1.33)	$14,178	$8,681 (0.61)
33-34 wGA	$22,484 (650)	$15,839 (1,326)	$9,450	$28,772 (276)	$19,931 (601)	$12,423	$26,194 (1.44)	$18,253	$11,204 (0.61)
31-32 wGA	$27,561 (273)	$17,766 (642)	$10,519	$44,254 (75)	$23,782 (235)	$14,186	$37,410 (1.76)	$21,315	$12,683 (0.59)
29-30 wGA	$22,819 (138)	$16,891 (381)	$13,524	$36,773 (37)	$34,032 (120)	$32,810	$31,052 (1.15)	$27,004	$24,903 (0.92)
< 29 wGA	$44,896 (80)	$39,354 (610)	$38,517	$44,201 (6)	$40,813 (177)	$40,694	$44,486 (1.11)	$40,215	$39,802 (0.99)
**Non-high-risk, 12-24 months old**									
All GA	Not Reported								
**High-risk, <2 years old**									
CHD	Not Reported								
CLD	Not Reported								
**Source**	**[5]**		**Calculated**	**[5]**		**Calculated**	**Calculated**		

Abbreviations: CA, chronological age; CHD, congenital heart disease; CLD, chronic lung disease; GA, gestational age; RSV-H, respiratory syncytial virus hospitalization with or without admission to intensive care unit; wGA, weeks gestational age.

Table S3: RSV-noICU costs per visit (unstratified by CA)

GA/high-risk condition	Medicaid costs in 2024 USD (in 2021 USD)	Commercial costs in 2024 USD (in 2021 USD)	Average costs in 2024 USD
**Non-high-risk, <1 year old**			
≥ 37 wGA	$6,193 ($5,720)	$12,285 ($11,347)	$9,787
35-36 wGA	$4,550 ($4,203)	$13,356 ($12,336)	$9,746
32-34 wGA	$10,251 ($9,468)	$14,995 ($13,850)	$13,050
29-31 wGA	$8,908 ($8,228)	$26,569 ($24,540)	$19,328
< 29 wGA	$13,293 ($12,278)	$27,444 ($25,348)	$21,642
**Non-high-risk, 12-23 months-old**			
All GA	$9,733 ($8,990)	$16,272 ($15,029)	$13,591
**High-risk, <1 year old[A]**			
CHD	$10,216 ($9,436)	$17,435 ($16,104)	$14,476
CLD	$11,507 ($10,628)	$22,461 ($20,746)	$17,970
**Source**	**[2]**	**[1]**	**Calculated**

Abbreviations: CA, chronological age; CHD, congenital heart disease; CLD, chronic lung disease; GA, gestational age; RSV-noICU, respiratory syncytial virus hospitalization without admission to intensive care unit; wGA, weeks gestational age.

[A] High-risk 12-23-month-olds were assumed to have the same RSV-noICU costs as <1-year-olds (due to lack of data).

Table S4: RSV-noICU costs per visit (stratified by CA and GA; 2024 USD)

GA/high-risk condition	Average costs	<3 months (multiplier)	3-11 months (multiplier)
**Non-high-risk, <1 year old**			
≥ 37	$9,787	$10,898 (1.11)	$8,537 (0.87)
35-36	$9,746	$12,971 (1.33)	$5,967 (0.61)
32-34	$13,050	$19,520 (1.50[A])	$7,963 (0.61 [A])

29-31	$19,328	$31,163 (1.61[B])	$12,992 (0.67 [B])
< 29	$21,642	$23,941 (1.11)	$21,420 (0.99)
**Non-high-risk, 12-23 months-old**			
All GA	$13,951	Costs not stratified by CA due to insufficient data	
**High-risk, <1 year old[C]**			
CHD	$14,476	Costs not stratified by CA due to insufficient data	
CLD	$17,970	Costs not stratified by CA due to insufficient data	
**Source**	**Table S3, Calculated**		

Abbreviations: CA, chronological age; CHD, congenital heart disease; CLD, chronic lung disease; GA, gestational age; RSV-H, respiratory syncytial virus hospitalization with or without admission to intensive care unit; wGA, weeks gestational age.

[A] Computed by taking the weighted average of multipliers for GA groups 33-34 wGA (weight = 2.09% = size of the 33-34 wGA birth cohort) and 31-32 wGA (weight = 0.49% = size of the 32 wGA birth cohort) with weights corresponding to the relative size of the birth cohort by GA [6].

[B] Computed by taking the weighted average of multipliers for GA groups 31-32 wGA (weight = 0.81% = size of the 31-32 wGA birth cohort) and 29-30 wGA (weight = 0.25% = size of the 30 wGA birth cohort) with weights corresponding to the relative size of the birth cohort by GA [6].

[C] High-risk 12-23-month-olds were assumed to have the same RSV-noICU costs as <1-year-olds (due to lack of data).

2.2 RSV-ICU costs

The costs of RSV-ICU hospitalization for infants covered by Medicaid and commercial insurance in 2021 USD were obtained from Choi and colleagues and Tran and colleagues, respectively, and inflated to 2024 USD [1,2,4]. The costs by healthcare setting were available separately for Medicaid and commercially insured patients. As in the previous section, a weighted average of the Medicaid and commercial insurance costs was used to estimate the RSV-ICU cost (**Table S5**). RSV-ICU costs were not stratified by CA.

Table S5: RSV-ICU costs per hospitalization

GA/high-risk condition	Medicaid costs in 2024 USD (in 2021 USD)	Commercial costs in 2024 USD (in 2021 USD)	Average costs in 2024 USD
**Non-high-risk, <1 year old**			
≥ 37 wGA	$23,681 ($21,873)	$37,636 ($34,762)	$31,915
35-36 wGA	$18,229	$51,218	$37,693

	($16,837)	($47,307)	
32-34 wGA	$25,028<br>($23,117)	$45,831<br>($42,331)	$37,302
29-31 wGA	$22,942<br>($21,190)	$88,485<br>($81,728)	$61,612
< 29 wGA	$45,585<br>($42,104)	$65,581<br>($60,573)	$57,383
**Non-high-risk, 12-23 months-old**			
All GA	$25,458<br>($23,514)	$35,381<br>($32,680)	$31,313
High-risk, <1 year old[A]			
CHD	$41,698<br>($38,514)	$46,901<br>($43,319)	$44,768
CLD	$42,010<br>($38,802)	$66,399<br>($61,329)	$56,400
**Source**	**[2]**	**[1]**	**Calculated**

Abbreviations: CHD, congenital heart disease; CLD, chronic lung disease; GA, gestational age; RSV-ICU, respiratory syncytial virus-Hospitalization-ICU; wGA, weeks gestational age.
[A] High-risk 12-23 months -olds were assumed to have the same RSV-ICU costs as <1-year-olds (due to lack of data).

2.3 RSV-ED and RSV-O costs

As with RSV-noICU and RSV-ICU, costs for RSV-ED ($1,207 in 2021 USD; inflated to $1,306 in 2024 USD) and RSV-O ($193 in 2021 USD; inflated to $209 in 2024 USD) outcomes for <12-month-olds were estimated by taking a weighted average of Medicaid and commercial costs obtained from Choi and colleagues and from Tran and colleagues (**Table S6**) [1,2,4]. These inputs were not stratified by GA, CA, or by high-risk status. Costs for 12-23-month-olds were similar in magnitude to costs for <1-year olds, therefore, costs for 12-23-month-olds were assumed to be equal to costs for infants.

Table S6: RSV-ED and RSV-O costs per episode

Outcome	Medicaid costs in 2024 USD (in 2021 USD)	Commercial costs in 2024 USD (in 2021 USD)	Average costs in 2024 USD
RSV-ED	$59.2<br>($482)	$1,851<br>($1,710)	$1,306
RSV-O	$157<br>($145)	$246<br>($227)	$209
**Source**	**[2]**	**[1]**	**Calculated**

Abbreviations: RSV-ED, respiratory syncytial virus emergency department visit; RSV-O, respiratory syncytial virus physician office visit.

2.4 Palivizumab costs

Dosage, drug cost, and the average weight of infants by GA and CA in months (weight-based dosing) for palivizumab were based on publicly available data [7,8]. The recommended palivizumab dose of 15 mg per kg using a 50 mg vial was assumed for the calculations. The cost per vial for palivizumab was assumed to be $1,228 [9]. Palivizumab was administered monthly during the RSV season for up to five doses. For example, a <29 wGA infant born in January would have palivizumab administered three times (i.e., from birth until the end of the RSV season: January, February, and March). On average, <29 wGA infants weighed 0.76 kg, 1.24 kg, and 1.98 kg in their first, second, and third months, and therefore, they would require a dose of palivizumab of at least 0.76kg * 15mg, 1.24kg * 15 mg, and 1.98kg * 15 mg, respectively. Equivalently, since each vial of palivizumab was assumed to contain a dose of 50 mg, they would require one vial of palivizumab in each of their first three months of life. Since CHD and CLD infants were not stratified by GA, it was conservatively assumed that palivizumab dosing was the same for CHD/CLD infants as for <29 wGA infants.

S3. Scenario 2: Alternative MALRI costs and ICU admission incidence

In this scenario, alternative cost data (obtained from the literature) to evaluate the health and economic impact of clesrovimab were used. Specifically, the hospitalization costs were obtained from McLaurin and colleagues [5]. The costs of RSV-ICU hospitalization were also obtained from McLaurin and colleagues and inflated to 2024 USD. The costs by healthcare setting were available separately for Medicaid and commercially insured patients. A weighted average of the Medicaid and commercial insurance costs was used to estimate the average annual cost of RSV-ICU hospitalization (**Table S7**). The percentage admitted to the ICU was also varied concurrently (**Table S8**), using values from the same source [5].

The annual costs for RSV-ED ($501 in 2020 USD; inflated to $552.99 in 2024 USD) and RSV-O ($73 in 2020 USD; inflated to $80.58 in 2024 USD) were also varied in this scenario and were obtained from the literature [10]. They were assumed to be the same for all GA groups due to a lack of GA-specific data.

Table S7: RSV-ICU costs per hospitalization (2024 USD)

Gestational age (weeks)	Medicaid	Commercial	Gestational age (weeks)	Weighted average[A]
≥ 37	$46,873	$47,190	≥ 37	$47,060
35-36	$49,602	$60,573	35-36	$56,075
33-34	$57,746	$78,272	32-34[B]	$70,523
31-32	$63,900	$79,854	29-31[C]	$82,629

29-30	$46,688	$119,357	<29	$89,939
< 29	$118,644	$69,992		
**Source**	**[5]**		**Source**	**Calculated**

Abbreviations: RSV-ICU, respiratory syncytial virus hospitalization with intensive care unit admission

[A] Weighted average computed with weights (Medicaid) 41% and (Commercial) 59%.

[B] Computed by taking the weighted average of multipliers for GA groups 33-34 wGA (weight = 2.09% = size of the 33-34 wGA birth cohort) and 31-32 wGA (weight = 0.49% = size of the 32 wGA birth cohort) with weights corresponding to the relative size of the birth cohort by GA[6].

[C] Computed by taking the weighted average of multipliers for GA groups 31-32 wGA (weight = 0.81% = size of the 31-32 wGA birth cohort) and 29-30 wGA (weight = 0.25% = size of the 30 wGA birth cohort) with weights corresponding to the relative size of the birth cohort by GA [6].

Table S8: Percentage of RSV-H cases admitted to the ICU

Gestational age (weeks)	Fraction of RSV-H admitted to ICU (%)
<29	26.68
29-31	21.24
32-34	18.53
35-36	14.54
≥37	8.54
**Source**	**[5]**

Abbreviations: ICU, intensive care unit; RSV-H, respiratory syncytial virus hospitalization with or without intensive care unit admission.

Table S9: Parameter distributions for sensitivity analyses

Parameter	Distribution*	Mean	Standard Deviation	DSA Lower Bound	DSA Upper Bound	Reference	Notes
**Efficacy**							
Clesrovimab efficacy	Beta	0.8720	0.0498[A]	0.7510	0.9340	[11,12]	Efficacy was estimated to be 87.2% (95% CI: 75.1% - 93.4%).
Palivizumab efficacy	Beta	0.5100	0.0692[A]	0.3700	0.6400	[13]	Efficacy was estimated to be 51% (95% CI: 37% - 64%)
RSVpreF vaccine efficacy	Beta	0.5130	0.0844[A]	0.2940	0.6680	[14]	Efficacy was estimated to be 51.3% (97.58% CI: 29.4% - 66.8%)
Nirsevimab efficacy (months 1-5)	Beta	0.7900	0.0461[A]	0.6850	0.8610	[15-17]	Efficacy was estimated to be 79.0% (Range for sensitivity analysis in CDC/UM model: 68.5% - 86.1%)
**Uptake**							
Clesrovimab uptake	Beta	0.5000	0.0500	0.4000[B]	0.6000[B]	Assumption	Standard deviation was assumed to be 10% of the mean
Palivizumab uptake	Beta	0.5000	0.0500	0.4000[B]	0.6000[B]	Assumption	
RSVpreF vaccine uptake	Beta	0.5000	0.0500	0.4000[B]	0.6000[B]	Assumption	
Nirsevimab uptake	Not applicable						Assumed uptake was equal to clesrovimab
**Intervention acquisition cost (per dose)**							
Clesrovimab	Gamma	$485	48.5	$395[B]	$585[B]	Assumption	Standard deviation was assumed to be 10% of the mean
Palivizumab	Gamma	$1,228	122.8	$999[B]	$1,480[B]	[9]	
RSVpreF vaccine	Gamma	$268	26.8	$218[B]	$323[B]	[18,19]	
Nirsevimab	Gamma	$485	48.5	$395[B]	$585[B]	Assumption	
**Quality-adjusted life-year (QALY) loss**							
QALY loss hospitalization (child)	Gamma	0.0169	0.00169	0.014[B]	0.02[B]	[20]	Standard deviation was assumed to be 10% of the mean
QALY loss ED (child)	Gamma	0.0134	0.00134	0.011[B]	0.016[B]		
QALY loss outpatient (child)	Gamma	0.0085	0.00085	0.007[B]	0.010[B]		
QALY loss hospitalization(caregiver)	Gamma	0.0066	0.00066	0.005[B]	0.008[B]		
QALY loss ED (caregiver)	Gamma	0.0069	0.00069	0.006[B]	0.008[B]		
QALY loss outpatient (caregiver)	Gamma	0.0041	0.00041	0.003[B]	0.005[B]		
QALY loss (premature death, <1 year)	Gamma	27.09	2.71	22.04[B]	32.65[B]	[21,22]	
QALY loss (premature death, 12-23 months-old)	Gamma	26.17	2.62	21.30[B]	31.55[B]		

Parameter	Distribution*	Mean	Standard Deviation	DSA Lower Bound	DSA Upper Bound	Reference	Notes
**Treatment costs**							
RSV-noICU, <3 months							
≥37 wGA	Gamma	$10,898	419[C]	$10,093[B]	$11,734[B]	[1,2,4,5]	SE (Medicaid): $\sqrt{8780^2/119}$ SE (commercial): $\sqrt{(10664^\wedge 2+101^\wedge 2)/33}$
35-36 wGA	Gamma	$12,971	546[C]	$11,924[B]	$14,062[B]		SE (Medicaid): $\sqrt{3793^\wedge 2/140}$ SE (commercial): $\sqrt{(8490^\wedge 2+49^\wedge 2)/199}$
32-34 wGA	Gamma	$19,520	2,872[C]	$14,302[B]	$25,538[B]		SE (Medicaid): $\sqrt{71804^\wedge 2/298}$ SE (commercial): $\sqrt{(15387^\wedge 2+169^\wedge 2)/34}$
29-31 wGA	Gamma	$31,163	8,426[C]	$16,899[B]	$49,719[B]		SE (Medicaid): $\sqrt{16723^\wedge 2/78}$ SE (commercial): $\sqrt{(68033^\wedge 2+688^\wedge 2)/71}$
<29 wGA	Gamma	$23,940	5,165[C]	$14,910[B]	$35,076[B]		SE (Medicaid): $\sqrt{22064^\wedge 2/84}$ SE (commercial): $\sqrt{(53252^\wedge 2+111^\wedge 2)/56}$
RSV-noICU, 3-11 months							
≥37 wGA	Gamma	$8,537	328[C]	$7,906[B]	$9,192[B]	[1,2,4,5]	SE (Medicaid): $\sqrt{8780^2/119}$ SE (commercial): $\sqrt{(10664^\wedge 2+101^\wedge 2)/33}$
35-36 wGA	Gamma	$5,967	251[C]	$5,485[B]	$6,469[B]		SE (Medicaid): $\sqrt{3793^\wedge 2/140}$ SE (commercial): $\sqrt{(8490^\wedge 2+49^\wedge 2)/199}$
32-34 wGA	Gamma	$7,963	1,172[C]	$5,834[B]	$10,418[B]		SE (Medicaid): $\sqrt{71804^\wedge 2/298}$ SE (commercial): $\sqrt{(15387^\wedge 2+169^\wedge 2)/34}$
29-31 wGA	Gamma	$12,992	3,513[C]	$7,046[B]	$20,728[B]		SE (Medicaid): $\sqrt{16723^\wedge 2/78}$ SE (commercial): $\sqrt{(68033^\wedge 2+688^\wedge 2)/71}$
<29 wGA	Gamma	$21,420	4,622[C]	$13,340[B]	$31,383[B]		SE (Medicaid): $\sqrt{22064^\wedge 2/84}$ SE (commercial): $\sqrt{(53252^\wedge 2+111^\wedge 2)/56}$
RSV-noICU, CHD, CLD, and-12-23-months-old							

Parameter	Distribution*	Mean	Standard Deviation	DSA Lower Bound	DSA Upper Bound	Reference	Notes
CHD (<2-years)	Gamma	$14,476	1,465 [C]	$11,747 [B]	$17,484 [B]	[1,2,4]	SE (Medicaid): $\sqrt{12367^2/32}$ SE (commercial): $\sqrt{(11634^2+595^2)/46}$
CLD (<2-years)	Gamma	$17,970	3,126 [C]	$12,373 [B]	$24,595 [B]		SE (Medicaid): $\sqrt{20178^2/109}$ SE (commercial): $\sqrt{(42089^2+568^2)/80}$
Healthy 12-23-month-olds	Gamma	$13,591	482 [C]	$12,663 [B]	$14,551 [B]		SE (Medicaid): $\sqrt{11056^2/116}$ SE (commercial): $\sqrt{(15585^2+141^2)/40}$
RSV-ICU, <12 months							
≥37 wGA	Gamma	$31,915	1,566 [C]	$28,919 [B]	$35,056 [B]	[1,2,4,5]	SE (Medicaid): $\sqrt{25379^2/69}$ SE (commercial): $\sqrt{(37013^2+63^2)/913}$
35-36 wGA	Gamma	$37,693	4,889 [C]	$28,725 [B]	$47,860 [B]		SE (Medicaid): $\sqrt{30884^2/53}$ SE (commercial): $\sqrt{(59926^2+52^2)/72}$
32-34 wGA	Gamma	$37,302	3,166 [C]	$31,354 [B]	$43,758 [B]		SE (Medicaid): $\sqrt{54808^2/143}$ SE (commercial): $\sqrt{(47890^2+73^2)/159}$
29-31 wGA	Gamma	$61,612	10,484 [C]	$42,802 [B]	$83,795 [B]		SE (Medicaid): $\sqrt{47540^2/56}$ SE (commercial): $\sqrt{(92173^2+216^2)/34}$
<29 wGA	Gamma	$57,383	10,452 [C]	$38,755 [B]	$79,607 [B]		SE (Medicaid): $\sqrt{69147^2/61}$ SE (commercial): $\sqrt{(78781^2+120^2)/27}$
RSV-ICU, CHD, CLD, and 12-23-month-olds							
CHD (<2-years)	Gamma	$44,768	$7,646 [C]	$31,054 [B]	$60,950 [B]	[1,2,4]	SE (Medicaid): $\sqrt{64721^2/28}$ SE (commercial): 8,429 (estimated by interpolating between RSV-H and RSV-noICU)
CLD (<2-years)	Gamma	$56,400	$7,756 [C]	$42,233 [B]	$72,584[B]		SE (Medicaid): $\sqrt{69616^2/76}$ SE (commercial): $\sqrt{(65686^2+221^2)/37}$

Parameter	Distribution*	Mean	Standard Deviation	DSA Lower Bound	DSA Upper Bound	Reference	Notes
Healthy 12-23-month-olds	Gamma	$31,313	$2,187 [C]	$27,174 [B]	$35,742 [B]		SE (Medicaid): $\sqrt{35647^2/61}$ SE (commercial): $\sqrt{(44699^2 + 75^2)/120}$[illegible]
RSV-ED and RSV-O							
RSV-ED, <1 year	Gamma	$1,306	20 [C]	$1,266 [B]	$1,347 [B]	[1,2,4]	SE (Medicaid): $\sqrt{476^2/1389}$ SE (commercial): $\sqrt{(3036^2 + 111^2)/970}$[illegible]
RSV-ED, 12-23 months-old	Gamma	$1,311	27 [C]	$1,259 [B]	$1,364 [B]		SE (Medicaid): $\sqrt{394^2/472}$ SE (commercial): $\sqrt{(2406^2 + 207^2)/362}$[illegible]
RSV-O, <1 year	Gamma	$210	2 [C]	$205 [B]	$214 [B]		SE (Medicaid): $\sqrt{190^2/6649}$ SE (commercial): $\sqrt{(650^2 + 350^2)/6042}$[illegible]
RSV-O, 12-23 months-old	Gamma	$216	7 [C]	$203 [B]	$229 [B]		SE (Medicaid): $\sqrt{159^2/1740}$ SE (commercial): $\sqrt{(1633^2 + 160^2)/252}$[illegible]
Other							
Births covered by Medicaid	Beta	41%	4.1%	33.1%	49.1%	[3]	Standard deviation was assumed to be 10% of the mean
**Incidence of RSV Disease**							
RSV-ED							
<1 month	Beta	1.96%	0.14% [A]	1.68%	2.24%	[23]	Incidence was estimated to be 1.96% (95% CI: 1.68% - 2.24%)
1 month	Beta	6.42%	0.47% [A]	5.49%	7.34%		Incidence was estimated to be 6.42% (95% CI: 5.49% - 7.34%)
2 months	Beta	7.24%	0.53% [A]	6.20%	8.29%		Incidence was estimated to be 7.24% (95% CI: 6.20% - 8.29%)
3 months	Beta	10.52%	0.77% [A]	9.01%	12.04%		Incidence was estimated to be 10.52% (95% CI: 9.01% - 12.04%)
4 months	Beta	11.60%	0.85% [A]	9.93%	13.27%		Incidence was estimated to be 11.60% (95% CI: 9.93% - 13.27%)
5 months	Beta	7.13%	0.52% [A]	6.11%	8.16%		Incidence was estimated to be 7.13% (95% CI: 6.11% - 8.16%)
6 months	Beta	8.18%	0.60% [A]	7.01%	9.36%		Incidence was estimated to be 8.18% (95% CI: 7.01% - 9.36%)
7 months	Beta	5.61%	0.41% [A]	4.80%	6.42%		Incidence was estimated to be 5.61% (95% CI: 4.80% - 6.42%)

Parameter	Distribution*	Mean	Standard Deviation	DSA Lower Bound	DSA Upper Bound	Reference	Notes
8 months	Beta	5.56%	0.40% A	4.76%	6.35%		Incidence was estimated to be 5.56% (95% CI: 4.76% - 6.35%)
9 months	Beta	5.56%	0.41% A	4.76%	6.36%		Incidence was estimated to be 5.56% (95% CI: 4.76% - 6.36%)
10 months	Beta	4.04%	0.30% A	3.46%	4.62%		Incidence was estimated to be 4.04% (95% CI: 3.46% - 4.62%)
11 months	Beta	5.56%	0.41% A	4.76%	6.36%		Incidence was estimated to be 5.56% (95% CI: 4.76% - 6.36%)
12 months	Beta	6.78%	0.52% A	5.76%	7.99%		Incidence was estimated to be 6.78% (95% CI: 5.76% - 7.99%)
13 months	Beta	5.04%	0.38% A	4.28%	5.79%		Incidence was estimated to be 5.04% (95% CI: 4.28% - 5.79%)
14 months	Beta	8.46%	0.64% A	7.19%	9.72%		Incidence was estimated to be 8.46% (95% CI: 7.19% - 9.72%)
15 months	Beta	3.97%	0.30% A	3.77%	4.56%		Incidence was estimated to be 3.97% (95% CI: 3.77% - 4.56%)
16 months	Beta	4.43%	0.34% A	3.77%	5.09%		Incidence was estimated to be 4.43% (95% CI: 3.77% - 5.09%)
17 months	Beta	8.24%	0.63% A	7.00%	9.47%		Incidence was estimated to be 8.24% (95% CI: 7.00% - 9.47%)
18 months	Beta	4.53%	0.34% A	3.86%	5.21%		Incidence was estimated to be 4.53% (95% CI: 3.86% - 5.21%)
19 months	Beta	4.59%	0.35% A	3.91%	5.28%		Incidence was estimated to be 4.59% (95% CI: 3.91% - 5.28%)
20 months	Beta	6.81%	0.52% A	5.79%	7.82%		Incidence was estimated to be 6.81% (95% CI: 5.79% - 7.82%)
21 months	Beta	2.93%	0.22% A	2.49%	3.36%		Incidence was estimated to be 2.93% (95% CI: 2.49% - 3.36%)
22 months	Beta	2.60%	0.20% A	2.21%	2.99%		Incidence was estimated to be 2.60% (95% CI: 2.21% - 2.99%)
23 months	Beta	5.23%	0.40% A	4.45%	6.02%		Incidence was estimated to be 5.23% (95% CI: 4.45% - 6.02%)
RSV-O							
<1 month	Beta	8.52%	0.72%	7.10%	9.93%	[23]	Incidence was estimated to be 8.52% (95% CI: 7.10% - 9.93%)
1 month	Beta	18.79%	1.59% A	15.66%	21.91%		Incidence was estimated to be 18.79% (95% CI: 15.66% - 21.91%)

Parameter	Distribution*	Mean	Standard Deviation	DSA Lower Bound	DSA Upper Bound	Reference	Notes
2 months	Beta	23.42%	1.99% A	19.52%	27.31%		Incidence was estimated to be 23.42% (95% CI: 19.52% - 27.31%)
3 months	Beta	23.26%	1.97% A	19.40%	27.13%		Incidence was estimated to be 23.26% (95% CI: 19.40% - 27.13%)
4 months	Beta	26.50%	2.41% A	22.10%	30.91%		Incidence was estimated to be 26.50% (95% CI: 22.10% - 30.91%)
5 months	Beta	28.92%	2.61% A	24.11%	33.72%		Incidence was estimated to be 28.92% (95% CI: 24.11% - 33.72%)
6 months	Beta	26.47%	2.41% A	22.07%	30.87%		Incidence was estimated to be 26.47% (95% CI: 22.07% - 30.87%)
7 months	Beta	20.72%	1.76% A	17.28%	24.17%		Incidence was estimated to be 20.72% (95% CI: 17.28% - 24.17%)
8 months	Beta	27.78%	2.52% A	23.17%	32.40%		Incidence was estimated to be 27.78% (95% CI: 23.17% - 32.40%)
9 months	Beta	22.72%	1.92% A	18.94%	26.49%		Incidence was estimated to be 22.72% (95% CI: 18.94% - 26.49%)
10 months	Beta	24.17%	2.05% A	20.15%	28.18%		Incidence was estimated to be 24.17% (95% CI: 20.15% - 28.18%)
11 months	Beta	25.81%	2.36% A	21.52%	30.10%		Incidence was estimated to be 25.81% (95% CI: 21.52% - 30.10%)
12 months	Beta	18.80%	2.01% A	15.26%	22.34%		Incidence was estimated to be 18.80% (95% CI: 15.26% - 22.34%)
13 months	Beta	18.83%	2.02% A	15.28%	22.38%		Incidence was estimated to be 18.83% (95% CI: 15.28% - 22.38%)
14 months	Beta	22.58%	2.25% A	18.32%	26.83%		Incidence was estimated to be 22.58% (95% CI: 18.32% - 26.83%)
15 months	Beta	20.42%	2.04% A	16.57%	24.27%		Incidence was estimated to be 20.42% (95% CI: 16.57% - 24.27%)

Parameter	Distribution*	Mean	Standard Deviation	DSA Lower Bound	DSA Upper Bound	Reference	Notes
16 months	Beta	19.11%	2.04% A	15.51%	22.71%		Incidence was estimated to be 19.11% (95% CI: 15.51% - 22.71%)
17 months	Beta	26.34%	2.60% A	21.38%	31.31%		Incidence was estimated to be 26.34% (95% CI: 21.38% - 31.31%)
18 months	Beta	15.09%	1.65% A	12.25%	17.94%		Incidence was estimated to be 15.09% (95% CI: 12.25% - 17.94%)
19 months	Beta	13.24%	1.46% A	10.75%	15.74%		Incidence was estimated to be 13.24% (95% CI: 10.75% - 15.74%)
20 months	Beta	17.04%	1.84% A	13.83%	20.25%		Incidence was estimated to be 17.04% (95% CI: 13.83% - 20.25%)
21 months	Beta	9.63%	1.20% A	7.82%	11.45%		Incidence was estimated to be 9.63% (95% CI: 7.82% - 11.45%)
22 months	Beta	11.14%	1.24% A	9.04%	13.24%		Incidence was estimated to be 11.14% (95% CI: 9.04% - 13.24%)
23 months	Beta	24.13%	2.39% A	19.58%	28.68%		Incidence was estimated to be 24.13% (95% CI: 19.58% - 28.68%)
Proportion of RSV-ED and RSV-O visits that are LRTIs							
RSV-ED, <6 months	Beta	65%	6.5%	52% B	77% B	[24]	Standard deviation was assumed to be 10% of the mean
RSV-ED, 6-11 months	Beta	50%	5.0%	40% B	60% B		
RSV-O, <6 months	Beta	65%	6.5%	52% B	77% B		
RSV-O, 6-11 months	Beta	30%	3.0%	24% B	36% B		
RSV-H							
<1 month	Beta	1.77%	0.40% A	1.54%	1.99%	[25]	Incidence was estimated to be 1.77% (95% CI: 1.54% - 1.99%)
1 month	Beta	3.12%	0.14% A	2.86%	3.41%		Incidence was estimated to be 3.12% (95% CI: 2.86% - 3.41%)
2 months	Beta	2.24%	0.43% A	2.04%	2.45%		Incidence was estimated to be 2.24% (95% CI: 2.04% - 2.45%)
3 months	Beta	1.57%	0.35% A	1.40%	1.74%		Incidence was estimated to be 1.57% (95% CI: 1.40% - 1.74%)
4 months	Beta	1.37%	0.31% A	1.20%	1.54%		Incidence was estimated to be 1.37% (95% CI: 1.20% - 1.54%)

Parameter	Distribution*	Mean	Standard Deviation	DSA Lower Bound	DSA Upper Bound	Reference	Notes
5 months	Beta	1.10%	0.29% [A]	0.96%	1.25%		Incidence was estimated to be 1.10% (95% CI: 0.96% - 1.25%)
6 months	Beta	0.96%	0.08% [A]	0.80%	1.11%		Incidence was estimated to be 0.96% (95% CI: 0.80% - 1.11%)
7 months	Beta	0.80%	0.22% [A]	0.64%	0.97%		Incidence was estimated to be 0.80% (95% CI: 0.64% - 0.97%)
8 months	Beta	0.74%	0.07% [A]	0.61%	0.88%		Incidence was estimated to be 0.74% (95% CI: 0.61% - 0.88%)
9 months	Beta	0.84%	0.08% [A]	0.69%	1.00%		Incidence was estimated to be 0.84% (95% CI: 0.69% - 1.00%)
10 months	Beta	0.60%	0.19% [A]	0.48%	0.72%		Incidence was estimated to be 0.60% (95% CI: 0.48% - 0.74%)
11 months	Beta	0.60%	0.19% [A]	0.48%	0.74%		Incidence was estimated to be 0.60% (95% CI: 0.48% - 0.74%)
12 months	Beta	0.63%	0.20%	0.50%	0.75%		Incidence was estimated to be 0.63% (95% CI: 0.50% - 0.75%)
13 months	Beta	0.50%	0.16%	0.39%	0.61%		Incidence was estimated to be 0.50% (95% CI: 0.39% - 0.61%)
14 months	Beta	0.58%	0.18%	0.46%	0.71%		Incidence was estimated to be 0.58% (95% CI: 0.46% - 0.71%)
15 months	Beta	0.54%	0.17%	0.43%	0.67%		Incidence was estimated to be 0.54% (95% CI: 0.43% - 0.67%)
16 months	Beta	0.40%	0.13%	0.29%	0.51%		Incidence was estimated to be 0.40% (95% CI: 0.29% - 0.51%)
17 months	Beta	0.37%	0.12%	0.27%	0.47%		Incidence was estimated to be 0.37% (95% CI: 0.27% - 0.47%)
18 months	Beta	0.37%	0.12%	0.28%	0.49%		Incidence was estimated to be 0.37% (95% CI: 0.28% - 0.49%)
19 months	Beta	0.34%	0.11%	0.25%	0.45%		Incidence was estimated to be 0.34% (95% CI: 0.25% - 0.45%)
20 months	Beta	0.28%	0.09%	0.20%	0.37%		Incidence was estimated to be 0.28% (95% CI: 0.20% - 0.37%)
21 months	Beta	0.21%	0.07%	0.14%	0.28%		Incidence was estimated to be 0.21% (95% CI: 0.14% - 0.28%)
22 months	Beta	0.18%	0.06%	0.12%	0.27%		Incidence was estimated to be 0.18% (95% CI: 0.12% - 0.27%)
23 months	Beta	0.29%	0.10%	0.20%	0.37%		Incidence was estimated to be 0.29% (95% CI: 0.20% - 0.37%)

Parameter	Distribution*	Mean	Standard Deviation	DSA Lower Bound	DSA Upper Bound	Reference	Notes
≥37 wGA	Beta	0.75%	0.02% A	0.72%	0.79%		Incidence was estimated to be 0.75% (95% CI: 0.72% - 0.79%)
35-36 wGA	Beta	1.13%	0.30% A	1.00%	1.28%		Incidence was estimated to be 1.13% (95% CI: 1.00% - 1.28%)
32-34 wGA	Beta	1.75%	0.14% A	1.48%	2.02%		Incidence was estimated to be 1.75% (95% CI: 1.48% - 2.02%)
29-31 wGA	Beta	1.93%	0.44% A	1.47%	2.37%		Incidence was estimated to be 1.93% (95% CI: 1.47% - 2.37%)
<29 wGA	Beta	2.49%	0.50% A	1.93%	3.06%		Incidence was estimated to be 2.49% (95% CI: 1.93% - 3.06%)
CHD	Beta	9.72% D	1.16% D	7.56% B	12.12% B	[26]	63 RSV-H were observed out of a sample size of 648
CLD	Beta	12.78% D	2.05% D	9.04% B	17.05% B	[27]	34 RSV-H were observed out of a sample size of 266
Probability of ICU admission							
≥37 wGA	Beta	27.60% D	1.49% D	24.73% B	30.56% B	[1,2]	Medicaid: 69 RSV-H were admitted to the ICU out of 188 Commercial: 913 RSV-H were admitted to the ICU out of 4,291
35-36 wGA	Beta	26.93% D	2.06% D	23.00% B	31.06% B		Medicaid: 53 RSV-H were admitted to the ICU out of 193 Commercial: 72 RSV-H were admitted to the ICU out of 271
32-34 wGA	Beta	31.76% D	1.52% D	28.82% B	34.78% B		Medicaid: 143 RSV-H were admitted to the ICU out of 441 Commercial: 159 RSV-H were admitted to the ICU out of 508
29-31 wGA	Beta	36.24% D	3.21% D	30.07% B	42.64% B		Medicaid: 56 RSV-H were admitted to the ICU out of 134 Commercial: 34 RSV-H were admitted to the ICU out of 105
<29 wGA	Beta	36.44% D	3.47% D	29.79% B	43.36% B		Medicaid: 61 RSV-H were admitted to the ICU out of 145 Commercial: 27 RSV-H were admitted to the ICU out of 83
CHD (<2 years)	Beta	37.01% D	1.16% D	28.88% B	45.53% B		Medicaid: 28 RSV-H were admitted to the ICU out of 60 Commercial: 20 RSV-H were admitted to the ICU out of 66

Parameter	Distribution*	Mean	Standard Deviation	DSA Lower Bound	DSA Upper Bound	Reference	Notes
CLD (<2 years)	Beta	35.50% D	2.05% D	29.85% B	41.36% B		Medicaid: 76 RSV-H were admitted to the ICU out of 185 Commercial: 37 RSV-H were admitted to the ICU out of 117
Healthy 12-23-month-olds	Beta	25.83% D	1.60% D	22.75% B	29.03% B		Medicaid: 61 RSV-H were admitted to the ICU out of 177 Commercial: 257 RSV-H were admitted to the ICU out of 1,296
RSV case fatality ratio							
Non-high-risk	Beta	0.04%	0.004%	0.03% B	0.05% B		Standard deviation was assumed to be 10% of the mean value
High-risk	Beta	0.90%	0.090%	0.73% B	1.08% B		
**Productivity Loss**							
RSV-H	Gamma	59.2 h	5.92	48.2 h B	71.4 h B	[9]	Hutton reports 7.4 days average productivity loss. It was assumed eight hours per day and a standard deviation of 10% of the mean.
RSV-ED	Gamma	20.0 h	0.14	19.7 h B	20.3 h B	[28,29]	Bosis reports 2.5 days median productivity loss for RSV-ED.<br><br>Heikkinen reports a similar result for RSV-O (mean: 2.6 days; standard deviation: 1.5, sample size: 110).<br><br>It was assumed a mean of 2.5 days with a standard error of $1.5/\sqrt{110}$.
RSV-O	Gamma	20.0 h	0.14	19.7 h B	20.3 h B		
Average hourly wage	Gamma	$31.48	0.09	$31.30	$31.67	[30]	Relative standard error is reported to be 0.3%
Lifetime productivity at birth	Gamma	$1,914,459	191,445.9	$1,557,680	$2,307,477	[31]	Standard deviation was assumed to be 10% of the mean.
**Demographics**							
Birth cohort by GA and high-risk condition							
≥37 wGA	Dirichlet E	89.68%	Various F	N/A E	N/A E	[6]	Maximum relative standard deviation was observed to be 7.08%
35-36 wGA		5.95%					
32-34 wGA		2.57%					
29-31 wGA		0.72%					
<29 wGA		0.60%					
CHD		0.25%					
CLD		0.24%					
Births by month							
January	Dirichlet E	8.11%	Various F	N/A E	N/A E	[6]	Maximum relative standard deviation was observed to be 3.51%
February		7.55%					
March		8.29%					
April		7.93%					
May		8.32%					

Parameter	Distribution*	Mean	Standard Deviation	DSA Lower Bound	DSA Upper Bound	Reference	Notes
June		8.36%					
July		8.79%					
August		9.06%					
September		8.68%					
October		8.52%					
November		8.12%					
December		8.28%					
Force of infection							
January	Dirichlet E	25.00%	Various F			[32]	Maximum relative standard deviation was observed to be 44.0%
February		21.84%					
March		13.86%					
April		4.50%					
May		1.30%					
June		0.60%					
July		0.47%					
August		0.62%					
September		1.16%					
October		2.95%					
November		8.07%					
December		19.61%					

Abbreviations: CHD, congenital heart disease; CI, confidence interval; CLD, chronic lung disease; ICU, intensive care unit; LRTI, lower respiratory tract infection; QALY, quality-adjusted life-year; RSV, respiratory syncytial virus; RSV-ED, RSV emergency department visit; RSV-H, RSV hospitalization; RSV-noICU, RSV hospitalization without ICU admission; RSV-ICU, RSV hospitalization with ICU admission; RSV-O, RSV outpatient visit; wGA, weeks gestational age

*Estimated parameters are often approximately normal (for example, by the Central Limit Theorem). To ensure that randomly drawn parameter values remain within feasible regions (e.g., 0-1 for probabilities and >0 for costs) we do not use Normal distributions for our parameters. Instead of normal distributions we use closely related Beta and Gamma distributions. For parameters on the interval [0,1] we use the Beta distribution, while for parameters on the interval [0, ∞) we use the Gamma distribution, which are approximately normal under the right conditions. Dirichlet distributions are used in situations where parameters are between 0-1 and the order must be preserved [33].

A Standard deviation chosen to minimize difference between $\alpha/2$% and (1- $\alpha/2$)% quantiles and lower and upper bounds of (1-α)% confidence interval (CI).

B Lower bound is equal to the 2.5% quantile of the distribution used for the PSA. Upper bound is equal to the 97.5% quantile of the distribution used for the PSA.

C Tran and colleagues and Choi and colleagues report cost data for commercially insured and Medicaid covered infants, respectively. In addition to the sample average, sample size and standard deviation are reported (from which standard error can be computed). The inflation factor and CA multiplier was modeled as constants and assume that commercial and Medicaid costs are independent so that cost inputs are approximately Normally distributed with mean $inflation\ factor \times CA\ multiplier \times (41\% \times cost_{Medicaid} + 59\% \times cost_{commercial})$ and variance $(inflation\ factor \times CA\ multiplier)^2 \times (41\%^2 \times standardError^2_{Medicaid} + 59\%^2 \times standardError^2_{commercial})$. To avoid having to truncate a normal distribution (since treatment costs cannot be negative), in place of a Normal distribution, a Gamma distribution (with same mean and standard deviation as described above) was used.

D Tran and colleagues and Choi and colleagues report ICU admission data for commercially insured and Medicaid covered infants, respectively. The sample mean and variance using a MLE approach was estimated. Under this approach the sample mean should be approximately Normally distributed. Assuming independence, we approximate the weighted average by a Normal distribution with mean $(41\% \times ICUadmisionRate_{Medicaid} + 59\% \times ICUadmissionRate_{commercial})$ and variance $(41\%^2 \times variance_{Medicaid} + 59\%^2 \times variance_{comemrcial})$. We then approximate the Normal distribution by a Beta distribution (with mean and variance as described) in place of a Normal distribution to avoid having to truncate the distribution's support (the parameter must be between 0 and 1).

E DSA cannot be performed on Dirichlet distributed parameters, i.e., these parameters cannot be modified independently of each other as they must sum to 1.

[F] Dirichlet parameters are proportional to the mean values. The proportionality constant is chosen to minimize the maximum of the squared differences of the relative standard deviation (standard deviation divided by mean) between the distribution and the data.

S4. Results

Table S10: NNI to avert one MALRI outcome

Scenario	NNI		
	RSV-O	RSV-ED	RSV-H
**Clesrovimab versus nirsevimab**			
Comparison #1	70	199	658
S1.1: alternative waning and durations	112	305	930
S1.2: Alternative duration of protection	133	363	1037
S2: alternative cost data	70	199	658
S3: healthcare perspective	70	199	658
**Clesrovimab (for all infants) versus palivizumab (for high-risk-infants only)**			
Comparison #2	13	34	103
**Nirsevimab (for all infants) versus palivizumab (high-risk infants only)**			
Comparison #3	15	42	122
**Clesrovimab versus RSVpreF**			
Comparison #4	20	55	179

Abbreviations: MALRI, medically attended lower respiratory infection; MV, maternal vaccination; NNI, number needed to immunize; RSV, respiratory syncytial virus; RSV-ED, RSV emergency department visit; RSV-H, RSV hospitalization with or without intensive care unit admission; RSV-O, RSV office visit.

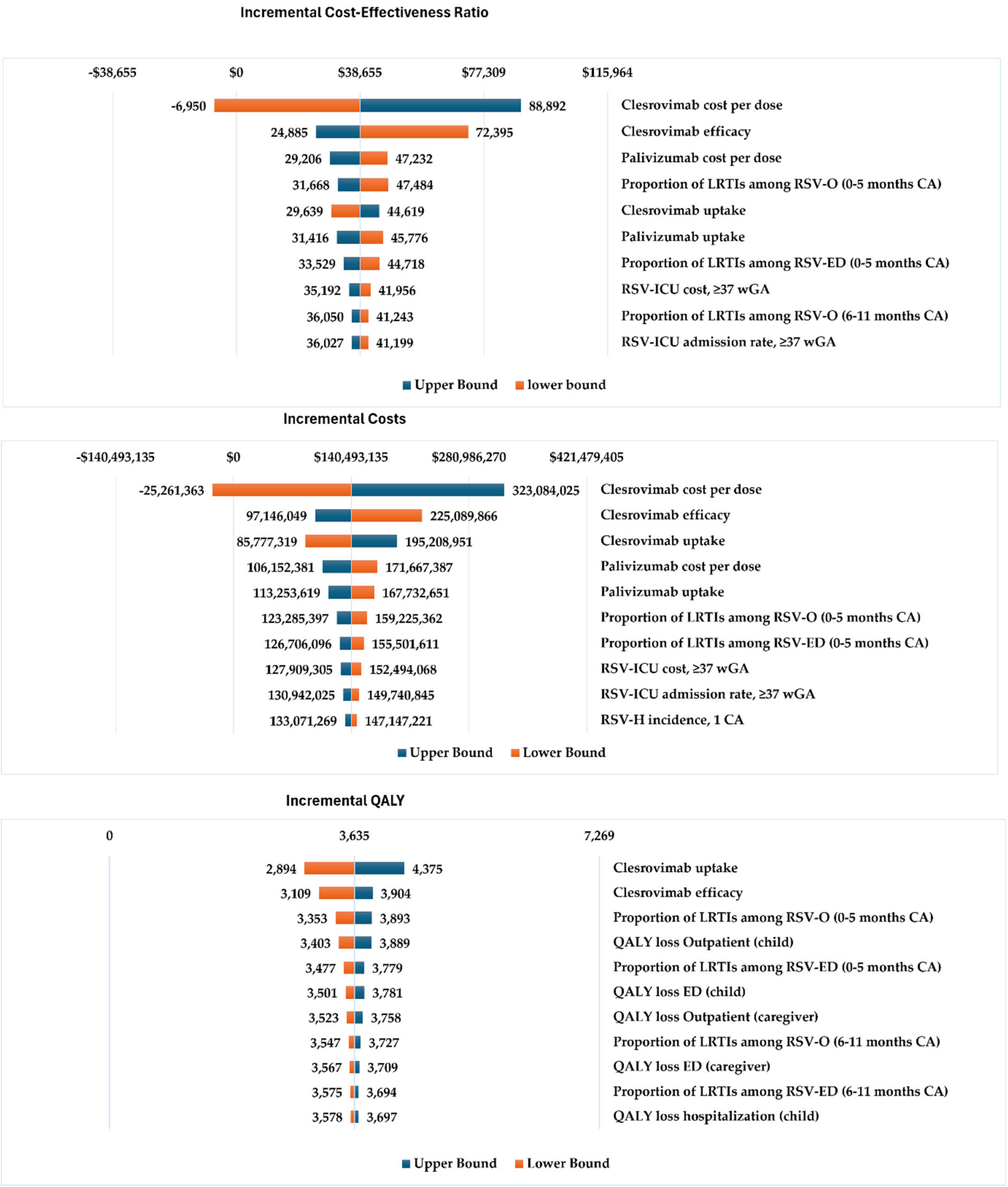

Figure S1: DSA of clesrovimab versus palivizumab showing the most influential inputs on the ICER (Top), Incremental costs (Middle) and Incremental QALYs (Bottom).

Abbreviations: CA, chronological age; LRTI, lower respiratory tract infection; QALY, quality-adjusted life-year; ED, RSV emergency department visit

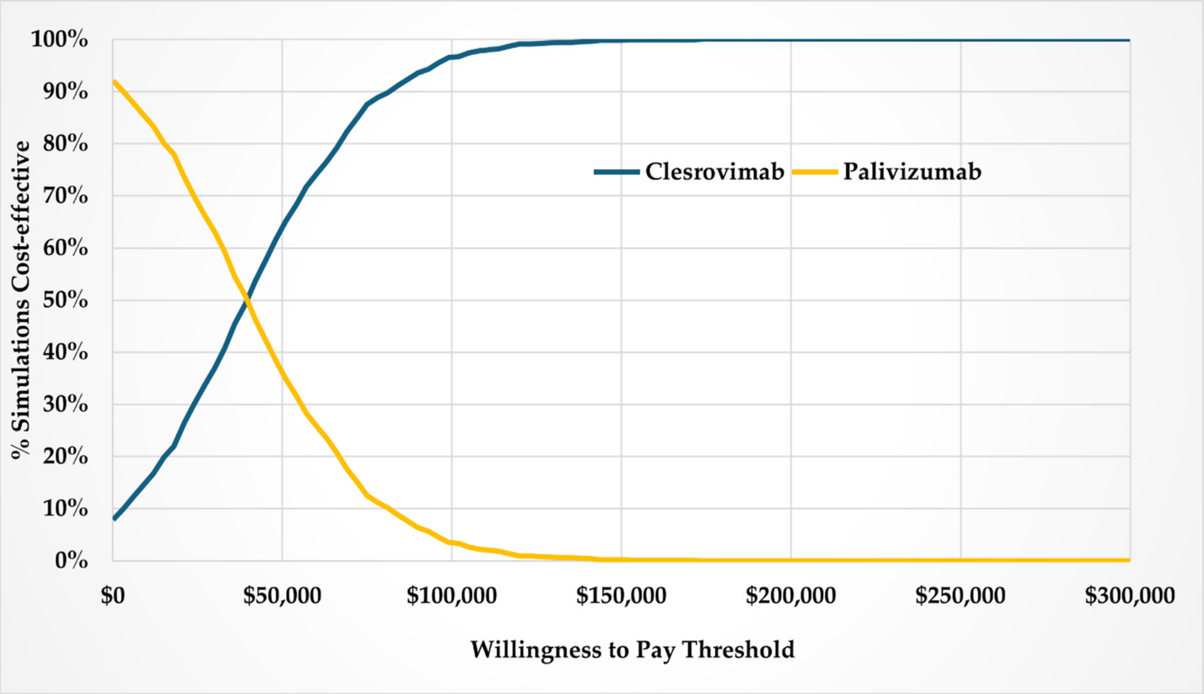

Figure S2: Cost-effectiveness acceptability curve for clesrovimab versus palivizumab (the proportion of PSA iterations in which the intervention is cost-effective).

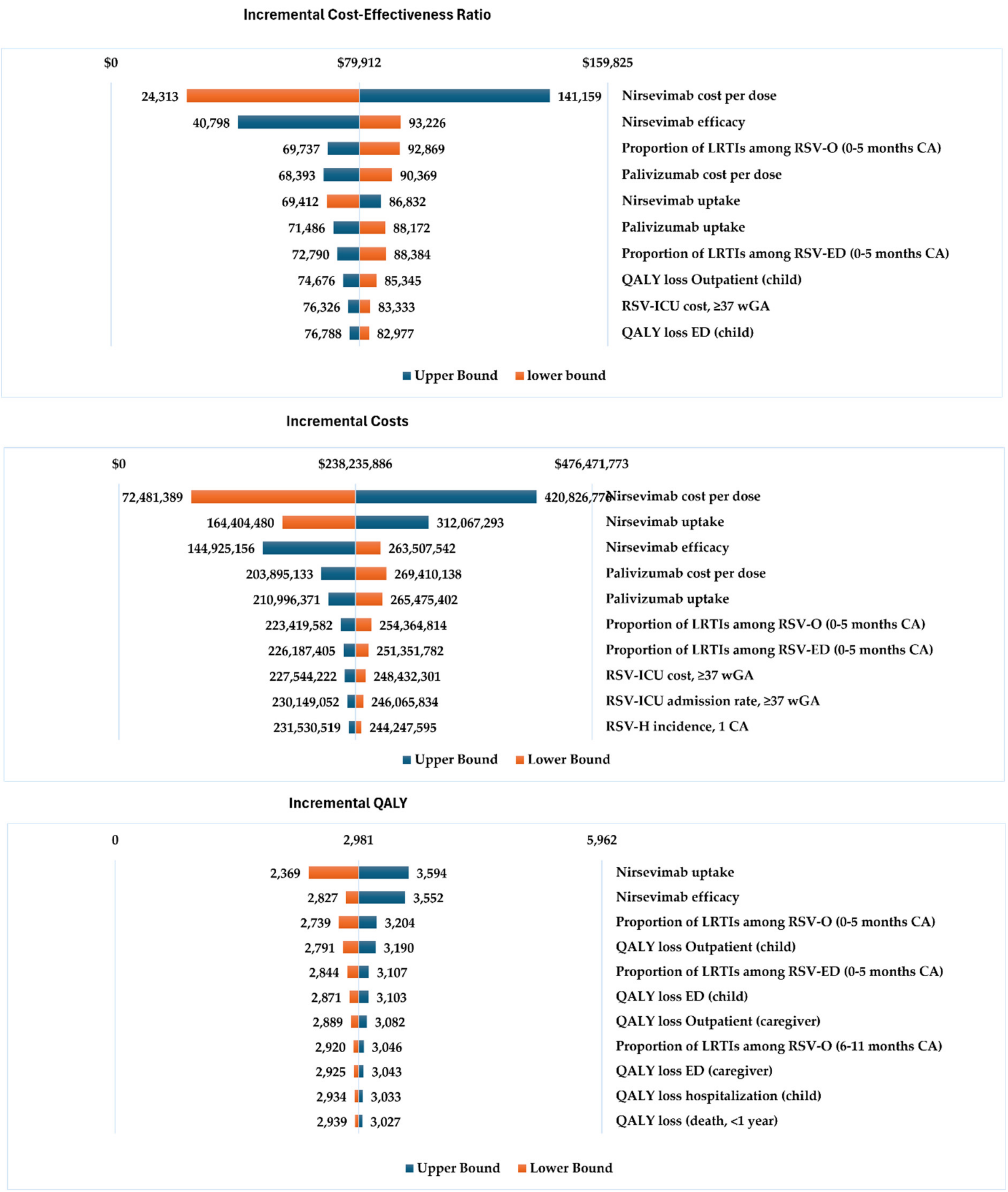

Figure S3: DSA of nirsevimab versus palivizumab showing the most influential inputs on the ICER (Top), Incremental costs (Middle) and Incremental QALYs (Bottom).

Abbreviations: CA, chronological age; LRTI, lower respiratory tract infection; QALY, quality-adjusted life-year; ED, RSV emergency department visit

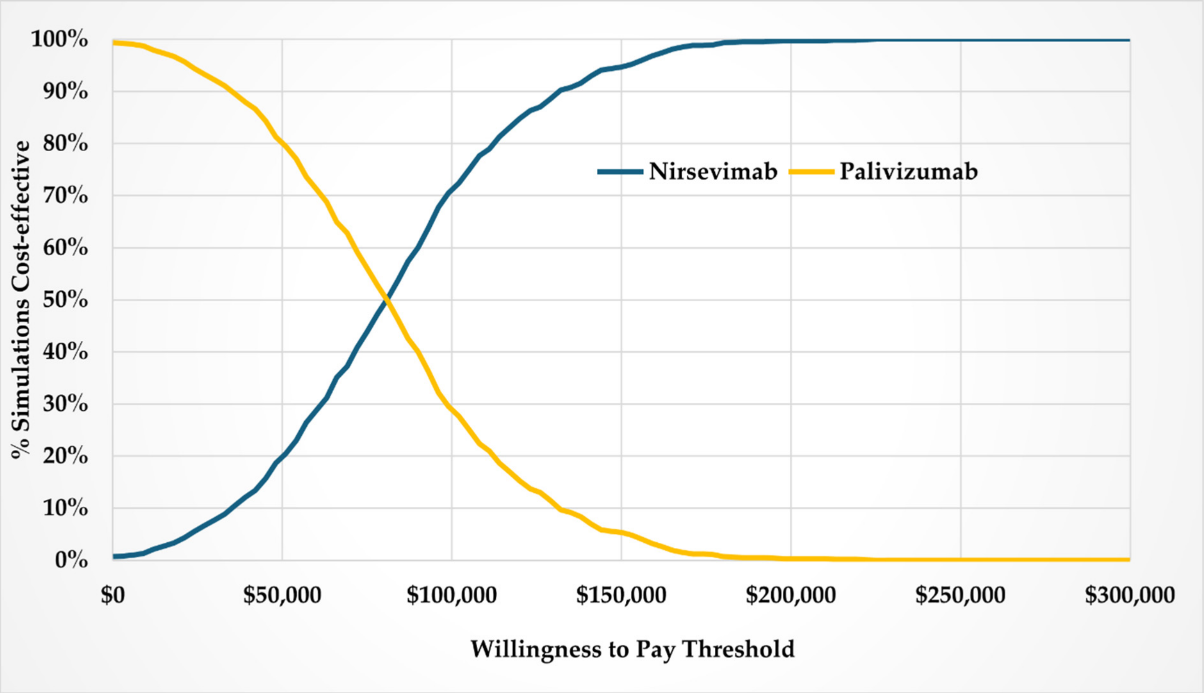

Figure S4: Cost-effectiveness acceptability curve for nirsevimab versus palivizumab (the proportion of PSA iterations in which the intervention is cost-effective).

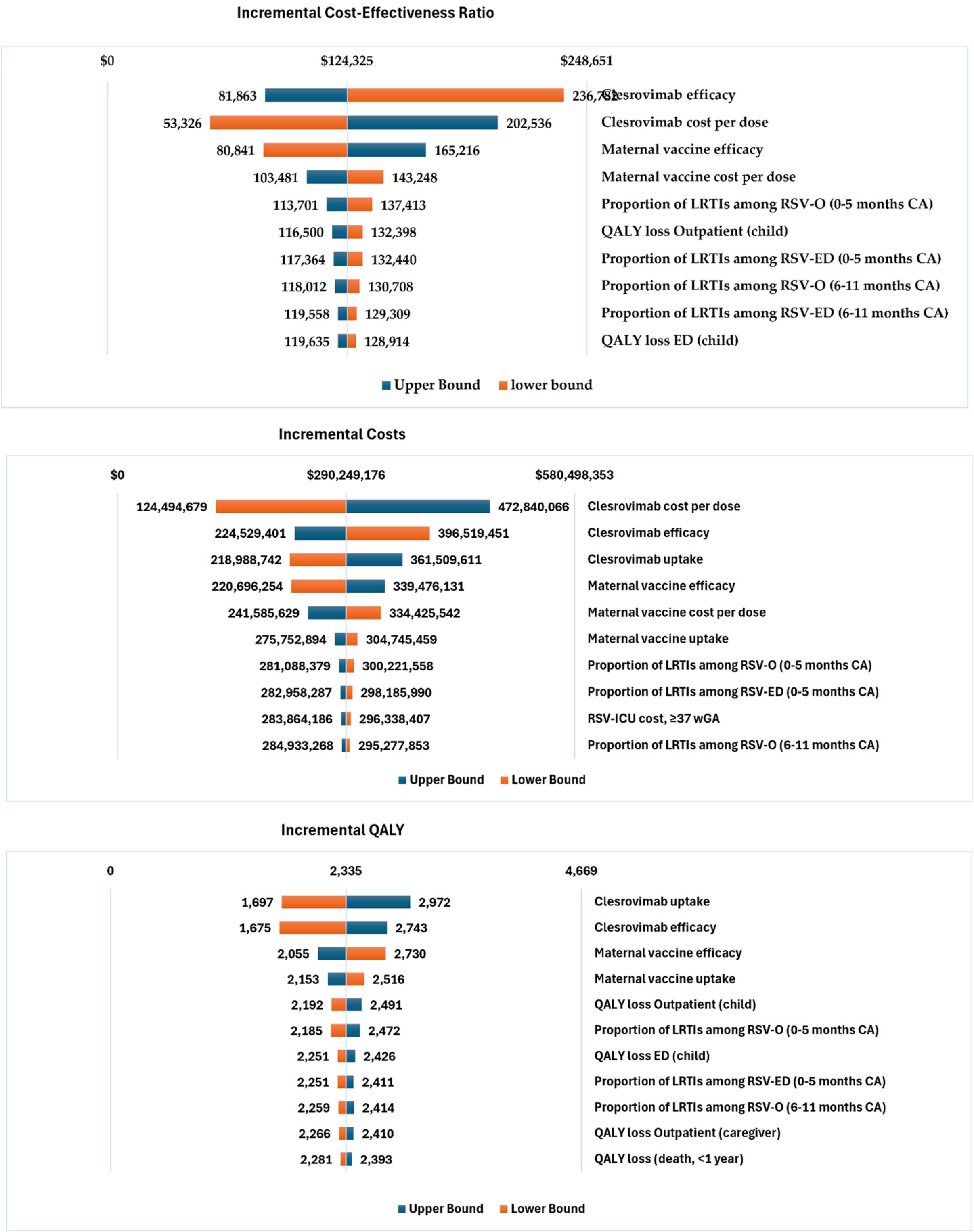

Figure S5: DSA of clesrovimab versus maternal vaccine showing the most influential inputs on the ICER (Top), Incremental costs (Middle) and Incremental QALYs (Bottom).

Abbreviations: CA, chronological age; LRTI, lower respiratory tract infection; QALY, quality-adjusted life-year; ED, RSV emergency department visit

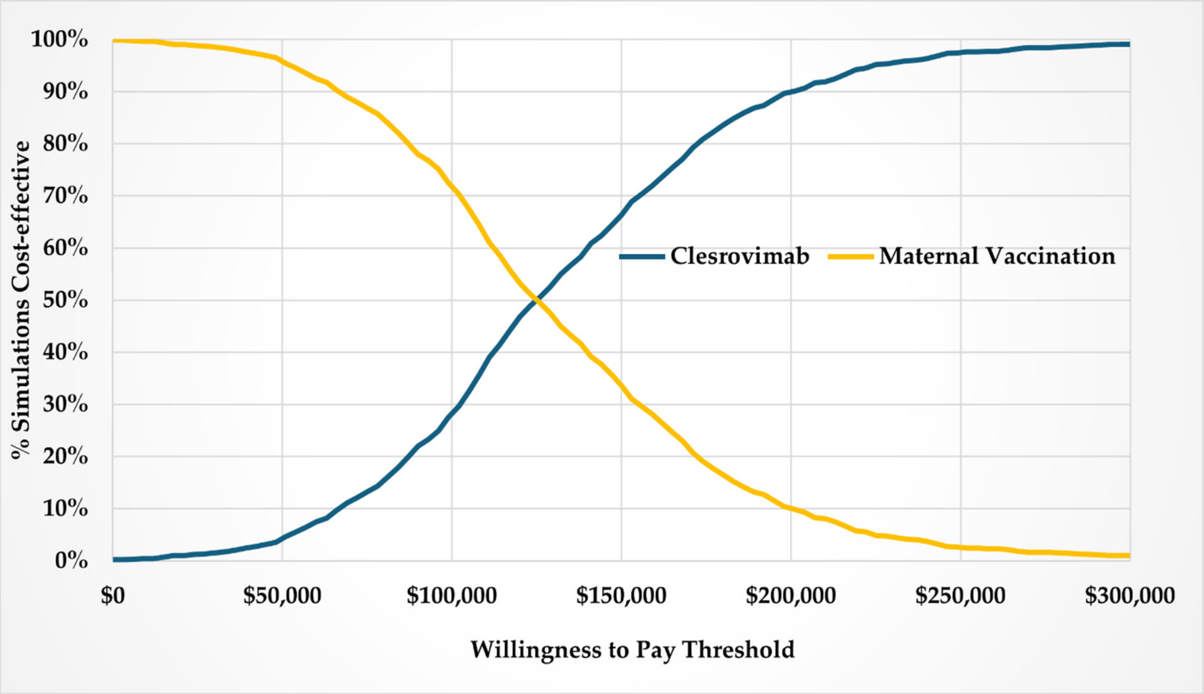

Figure S6: Cost-effectiveness acceptability curve for clesrovimab versus maternal vaccine (the proportion of PSA iterations in which the intervention is cost-effective).

References

1. Tran, P.T.; Nduaguba, S.O.; Wang, Y.; Diaby, V.; Finelli, L.; Choi, Y.; Winterstein, A.G. Economic Burden of Medically Attended Respiratory Syncytial Virus Infections Among Privately Insured Children Under 5 Years of Age in the USA. *Influenza Other Respir Viruses* **2024**, *18*, e13347, doi:10.1111/irv.13347.
2. Choi, Y.; Finelli, L. Cost of Medically Attended RSV Among Medicaid Beneficiaries ≤2 Years of Age by Underlying Risk Condition. *J Pediatric Infect Dis Soc* **2023**, *12*, 590-593, doi:10.1093/jpids/piad086.
3. Kaiser Family Foundation. Births Financed by Medicaid. Available online: https://www.kff.org/medicaid/state-indicator/births-financed-by-medicaid/?currentTimeframe=0&sortModel=%7B%22colId%22:%22Location%22,%22sort%22:%22asc%22%7D (accessed on September 19).
4. US Bureau of Labor Statistics. Medical care in U.S. city average, all urban consumers, not seasonally adjusted. Available online: https://data.bls.gov/series-report (accessed on September 18).

5. McLaurin, K.K.; Farr, A.M.; Wade, S.W.; Diakun, D.R.; Stewart, D.L. Respiratory syncytial virus hospitalization outcomes and costs of full-term and preterm infants. *J Perinatol* **2016**, *36*, 990-996, doi:10.1038/jp.2016.113.
6. National Center for Health Statistics. National Vital Statistics System, Natality on CDC WONDER Online Database. Data are from the Natality Records 2016-2022. Available online: http://wonder.cdc.gov/natality-expanded-current.html (accessed on September 17).
7. CDC National Center for Health Statistics. CDC Growth Charts. Available online: https://www.cdc.gov/growthcharts/clinical_charts.htm (accessed on October 22).
8. MedImmune. Synagis(R) (palivizumab) injection, for intramuscular use. Available online: https://www.accessdata.fda.gov/drugsatfda_docs/label/2017/103770s5200lbl.pdf (accessed on October 22).
9. Hutton, D.W. Economic Analysis of Nirsevimab in Pediatric Populations. Available online: https://www.cdc.gov/vaccines/acip/meetings/downloads/slides-2023-08-3/02-RSV-jones-508.pdf (accessed on August 29).
10. Bowser, D.M.; Rowlands, K.R.; Hariharan, D.; Gervasio, R.M.; Buckley, L.; Halasa-Rappel, Y.; Glaser, E.L.; Nelson, C.B.; Shepard, D.S. Cost of Respiratory Syncytial Virus Infections in US Infants: Systematic Literature Review and Analysis. *The Journal of Infectious Diseases* **2022**, *226*, S225-S235, doi:10.1093/infdis/jiac172.
11. Zar, H.J.; Simoes, E.A.F.; Madhi, S.A.; Ramilo, O.; Senders, S.D.; Shepard, J.S.; Laoprasopwattana, K.; Piedrahita, J.; Novoa, J.M.; Vargas, S.L.; et al. Clesrovimab for Prevention of RSV Disease in Healthy Infants. *N Engl J Med* **2025**, *393*, 1292-1303, doi:10.1056/NEJMoa2502984.
12. Zar, H.J.; Bont, L.J.; Manzoni, P.; Munoz, F.M.; Ramilo, O.; Chen, P.Y.; Novoa, J.M.; Ordonez, G.A.; Tsolia, M.; Tapiero, B.; et al. Clesrovimab in Infants and Children at Increased Risk for Severe RSV Disease. *N Engl J Med* **2025**, *393*, 1343-1345, doi:10.1056/NEJMc2506107.
13. Andabaka, T.; Nickerson, J.W.; Rojas-Reyes, M.X.; Rueda, J.D.; Bacic Vrca, V.; Barsic, B. Monoclonal antibody for reducing the risk of respiratory syncytial virus infection in children. *Cochrane Database Syst Rev* **2013**, Cd006602, doi:10.1002/14651858.CD006602.pub4.
14. Kampmann, B.; Madhi, S.A.; Munjal, I.; Simões, E.A.F.; Pahud, B.A.; Llapur, C.; Baker, J.; Pérez Marc, G.; Radley, D.; Shittu, E.; et al. Bivalent Prefusion F Vaccine in Pregnancy to Prevent RSV Illness in Infants. *N Engl J Med* **2023**, *388*, 1451-1464, doi:10.1056/NEJMoa2216480.
15. Simões, E.A.F.; Madhi, S.A.; Muller, W.J.; Atanasova, V.; Bosheva, M.; Cabañas, F.; Baca Cots, M.; Domachowske, J.B.; Garcia-Garcia, M.L.; Grantina, I.; et al. Efficacy of nirsevimab against respiratory syncytial virus lower respiratory tract infections in preterm and term infants, and pharmacokinetic extrapolation to infants with congenital heart disease and chronic lung disease: a pooled analysis of randomised controlled trials. *The Lancet Child & Adolescent Health* **2023**, *7*, 180-189, doi:https://doi.org/10.1016/S2352-4642(22)00321-2.
16. Sanofi, A.a. Nirsevimab for the prevention of RSV in all infants. ACIP presentation, October 2022., doi:https://stacks.cdc.gov/view/cdc/122373.
17. Jones, J.M.; Fleming-Dutra, K.E.; Prill, M.M.; Roper, L.E.; Brooks, O.; Sánchez, P.J.; Kotton, C.N.; Mahon, B.E.; Meyer, S.; Long, S.S.; McMorrow, M.L. Use of Nirsevimab for the Prevention of Respiratory Syncytial Virus Disease Among Infants and Young Children: Recommendations of the Advisory Committee on Immunization Practices — United States, 2023. *MMWR Morb Mortal Wkly Rep* **2023**, *72*, 920-925, doi:10.15585/mmwr.mm7234a4.
18. CDC Vaccines for Children Program. Current CDC Vaccine Price List. Available online: https://www.cdc.gov/vaccines-for-children/php/awardees/current-cdc-vaccine-price-list.html (accessed on October 22).
19. Hutton, D.W. Economic analysis of RSVpreF maternal vaccination. Available online: https://www.cdc.gov/acip/meetings/presentation-slides-september-22-2023.html (accessed on October 22).

20. Glaser, E.L.; Hariharan, D.; Bowser, D.M.; Gervasio, R.M.; Rowlands, K.R.; Buckley, L.; Nelson, C.B.; Shepard, D.S. Impact of Respiratory Syncytial Virus on Child, Caregiver, and Family Quality of Life in the United States: Systematic Literature Review and Analysis. *J Infect Dis* **2022**, *226*, S236-s245, doi:10.1093/infdis/jiac183.
21. Sullivan, P.W.; Lawrence, W.F.; Ghushchyan, V. A national catalog of preference-based scores for chronic conditions in the United States. *Med Care* **2005**, *43*, 736-749, doi:10.1097/01.mlr.0000172050.67085.4f.
22. Arias, E.; Xu, J.; Kochanek, K. United States Life Tables, 2021. *National Vital Statistics Reports* **2023**, *72*.
23. Lively, J.Y.; Curns, A.T.; Weinberg, G.A.; Edwards, K.M.; Staat, M.A.; Prill, M.M.; Gerber, S.I.; Langley, G.E. Respiratory Syncytial Virus–Associated Outpatient Visits Among Children Younger Than 24 Months. *Journal of the Pediatric Infectious Diseases Society* **2019**, *8*, 284-286, doi:10.1093/jpids/piz011.
24. Rainisch, G.; Adhikari, B.; Meltzer, M.I.; Langley, G. Estimating the impact of multiple immunization products on medically-attended respiratory syncytial virus (RSV) infections in infants. *Vaccine* **2020**, *38*, 251-257, doi:https://doi.org/10.1016/j.vaccine.2019.10.023.
25. Curns, A.T.; Rha, B.; Lively, J.Y.; Sahni, L.C.; Englund, J.A.; Weinberg, G.A.; Halasa, N.B.; Staat, M.A.; Selvarangan, R.; Michaels, M.; et al. Respiratory Syncytial Virus-Associated Hospitalizations Among Children <5 Years Old: 2016 to 2020. *Pediatrics* **2024**, *153*, doi:10.1542/peds.2023-062574.
26. Feltes, T.F.; Cabalka, A.K.; Meissner, H.C.; Piazza, F.M.; Carlin, D.A.; Top, F.H.; Connor, E.M.; Sondheimer, H.M.; for the Cardiac Synagis Study, G. Palivizumab prophylaxis reduces hospitalization due to respiratory syncytial virus in young children with hemodynamically significant congenital heart disease. *The Journal of Pediatrics* **2003**, *143*, 532-540, doi:https://doi.org/10.1067/S0022-3476(03)00454-2.
27. Palivizumab, a humanized respiratory syncytial virus monoclonal antibody, reduces hospitalization from respiratory syncytial virus infection in high-risk infants. The IMpact-RSV Study Group. *Pediatrics* **1998**, *102*, 531-537.
28. Bosis, S.; Esposito, S.; Niesters, H.G.; Crovari, P.; Osterhaus, A.D.; Principi, N. Impact of human metapneumovirus in childhood: comparison with respiratory syncytial virus and influenza viruses. *J Med Virol* **2005**, *75*, 101-104, doi:10.1002/jmv.20243.
29. Heikkinen, T.; Ojala, E.; Waris, M. Clinical and Socioeconomic Burden of Respiratory Syncytial Virus Infection in Children. *The Journal of Infectious Diseases* **2016**, *215*, 17-23, doi:10.1093/infdis/jiw475.
30. US Bureau of Labor Statistics. May 2023 National Occupational Employment and Wage Statistics: United States. Available online: https://www.bls.gov/oes/current/oes_nat.htm#00-0000 (accessed on August 29).
31. Grosse, S.D.; Krueger, K.V.; Pike, J. Estimated annual and lifetime labor productivity in the United States, 2016: implications for economic evaluations. *J Med Econ* **2019**, *22*, 501-508, doi:10.1080/13696998.2018.1542520.
32. Abdirizak, F.; Haynes, A. RSV Surveillance Data: 2010-2019. **2020**.
33. Andrew Briggs, K.C., Mark Sculpher. *Decision Modelling For Health Economic Evaluation*; Oxford University Press: 2006.
